# Malnourishment affects gene expression along the length of the small intestine

**DOI:** 10.3389/fnut.2022.894640

**Published:** 2022-09-02

**Authors:** Raquel M. Pinho, Lydia C. Garas, B. Carol Huang, Bart C. Weimer, Elizabeth A. Maga

**Affiliations:** ^1^Department of Animal Science, University of California, Davis, Davis, CA, United States; ^2^Department of Population Health and Reproduction, School of Veterinary Medicine, University of California, Davis, Davis, CA, United States

**Keywords:** malnourishment, intestinal gene expression, system biology, small intestine, childhood mortality

## Abstract

Malnourishment is a risk factor for childhood mortality, jeopardizing the health of children by aggravating pneumonia/acute respiratory infections and diarrheal diseases. Malnourishment causes morphophysiological changes resulting in stunting and wasting that have long-lasting consequences such as cognitive deficit and metabolic dysfunction. Using a pig model of malnutrition, the interplay between the phenotypic data displayed by the malnourished animals, the gene expression pattern along the intestinal tract, microbiota composition of the intestinal contents, and hepatic metabolite concentrations from the same animals were correlated using a multi-omics approach. Samples from the duodenum, jejunum, and ileum of malnourished (protein and calorie-restricted diet) and full-fed (no dietary restrictions) piglets were subjected to RNA-seq. Gene co-expression analysis and phenotypic correlations were made with WGCNA, while the integration of transcriptome with microbiota composition and the hepatic metabolite profile was done using mixOmics. Malnourishment caused changes in tissue gene expression that influenced energetic balance, cell proliferation, nutrient absorption, and response to stress. Repression of antioxidant genes, including glutathione peroxidase, in coordination with induction of metal ion transporters corresponded to the hepatic metabolite changes. These data indicate oxidative stress in the intestine of malnourished animals. Furthermore, several of the phenotypes displayed by these animals could be explained by changes in gene expression.

## Introduction

Malnourishment is a risk factor for childhood mortality, jeopardizing the health of children by aggravating pneumonia/acute respiratory infections and diarrheal diseases that combined accounted for 22.8% of the more than 1 million deaths of children under 5 years of age in 2017 (1). Malnutrition alone is linked to 45% of the total under-5 deaths (2). Appropriate translational animal models can aid in the study of this condition with respect to mechanisms underlying the physical state and the effect of different dietary interventions.

The use of swine models for human diseases is increasingly more common due to the anatomical, physiological, and size similarities of pigs to humans (3). To model diseases in children, piglets better resemble young developing children than rodents. Gastrointestinal similarities such as intestinal length: body weight ratio and digestive capabilities make it advantageous to use pig models in studies focusing on the intestinal tract, especially during early life (3, 4).

Malnourishment causes morphological and physiological changes leading to stunting and wasting that have long-lasting consequences, including cognitive deficit (5, 6), incomplete intestinal maturation (7, 8), and metabolic changes (9). Some of these effects are observed in the swine model for childhood undernutrition developed by Garas (10, 11). Morphologically, malnourished piglets had significant reductions in villi height and crypt depth and thinning of lamina propria along the length of the intestinal tract which was accompanied by physical decreases in size and weight of the individuals. Decreased barrier function in the jejunum was also observed, with transcellular and paracellular permeability being four times greater in the malnourished group compared with full-fed animals, indicating a potential vulnerability to invading bacteria. Furthermore, fecal and intestine contents of malnourished pigs had distinct microbiota composition from the full-fed animals (11). Overall, the model mimicked morphological and microbial changes observed in the intestines of people affected by malnourishment (12–16).

Transcriptomic data from the swine model can further the knowledge on the effects of malnutrition along the intestinal tract and contribute to the systems biology approach when integrated with the different omics and phenotypic data available for these animals. This systems biology approach allows for a more complete and nuanced analysis between molecular mechanisms and physical outcomes that could not be possible by analyzing individual biological layers.

Information about the molecular processes driving malnourished-induced morpho-physiological changes and the association between phenotypes and the large-scale genetic makeup of the host is still lacking. These associations could expose more precise and definitive conclusions about the crosstalk between genes and morphophysiological changes due to malnourishment. In this study, we investigated changes in gene expression along the length of the intestinal tract as a result of malnutrition and the association between gene expression and resulting phenotypes such as body weight, intestinal morphology and permeability, and microbiota composition.

## Materials and methods

### Animals, housing, and diet

The experimental design and the description of the animals, diets, and housing conditions were described previously (11). Briefly, at weaning, 21-day-old male pigs were fed a transition diet (21% protein, 5% fat *ad libitum*) for 4 days and then divided into two groups: one fed with a protein and calorie-restricted diet (14.2% protein deficient in lysine, 6% fat in restricted amount (3% body weight/day)—Mal) and the other fed with a standard grower diet *ad libitum* (18.7% protein, 6% fat-full-fed (FF)) for a period of 5 weeks. For the malnourished group, the feed was divided into two equal portions and fed twice daily. Body weight was measured every 2 days to monitor the response to the diet. After 5 weeks, the animals were necropsied and segments of the duodenum, jejunum, and ileum were collected and prepared for either histological analysis or RNA extraction as described (10, 11). Malnourished animals were on average half the final body weight of the FF animals, with significantly reduced weight gain, body length, body circumference, and Z-score. Furthermore, the group submitted to the restricted diet had a mean Z-score of−3.95 (11) indicating severe wasting and undernutrition. Intestinal contents from each section were also collected for microbial analysis by amplicon sequencing of the 16S bacterial ribosomal RNA gene (11). All tissue samples and phenotypic data (*n* = 4 per group) used in this study were derived from these animals. This study was approved by the UC Davis Institutional Animal Care and Use Committee (IACUC) under Association of Assessment and Accreditation of Laboratory Animal Care International (AAALAC) approved conditions.

### Library construction and sequencing for tissue RNA-seq

Total RNA was extracted from the duodenum, jejunum, and ileum with standard protocols previously described (11). The resulting total RNA was analyzed for integrity using the Agilent 2100 Bioanalyzer system (Agilent Technologies) and samples with an integrity number > 7 were used for library construction as previously described (17, 18). With an input of 2 μg of total RNA with the KAPA Stranded mRNA-Seq kit (Roche) and Agencourt^®^ AMPure^®^ reagent (Beckman Coulter) with custom adapters compatible with the Illumina platform (Integrated DNA Technologies—Supplementary Table 1), the samples were randomly pooled, seven to eight samples per lane, at a concentration of 5nM for 100 bp single-end sequencing across multiple lanes using the HiSeq 3000 System (Illumina, San Diego, CA) at the UC Davis Genome Center DNA Technologies Core (Davis, CA).

### RNA-seq data analysis

Raw reads from the total RNA-Seq for four biological replicates in each group were quality and adapter trimmed using Trimmomatic (version 0.33) (19). The transcript abundance count was done using both Salmon (v.0.91) (20) and Kallisto (v.0.43.1) (21) against the pig reference genome and coding sequence annotation (assembly Sscrofa11.1, GenBank assembly accession: GCA_000003025.6). The statistical analysis for differentially expressed (DE) transcripts was done using DESeq2 (v.1.22.2—Wald test (MLE) with betaPrior = FALSE) (21) and edgeR (v.3.24.3—glmQLFit) (22). Both DESeq2 and EdgeR models had a parameter accounting for unspecific variation, calculated based on the estimation made by RUVSeq (v.1.16.1) (23). Genes were considered significantly different when *p*-adjusted values (padj) were < 0.1 and absolute log_2_-fold change was at least 1.0 in both the DESeq2 and edgeR models with transcript counts from both Kallisto and Salmon. Annotation of the transcripts was done using the bioMart (24) package in R, and functional analysis was done using both the Gene Ontology (GO) and Reactome databases in R through RDAVIDWebService (v.1.20.0) (25) and ReactomePA (v.1.26.0) (26) packages, respectively. Using the GO analysis, pathways were considered significantly enriched if *p*-values were < 0.0001, and pathways with *p*-values < 0.05 were considered highlighted, and for reactome analysis, pathways were considered significant if padj < 0.05. The analysis of protein-protein interactions of DE genes was also done using STRING (27). The scripts used for this analysis are available at https://raquelpinho.github.io/RaquelPinho/.

### Validation of RNA-seq data

To validate sequencing results, quantitative real-time polymerase chain reaction (qRT-PCR) was carried out to measure the levels of key genes found to be DE. Total RNA was converted into cDNA as previously described (11). Expression levels of the genes C-X-C motif chemokine ligand 2 (CXCL2), phosphoenolpyruvate carboxykinase 1 (PCK1), malic enzyme 1 (ME1), dipeptidase 1 (DPEP1), and stearoyl-CoA desaturase (SCD) were quantified using TATA-box binding protein (TBP) as the housekeeping gene (Supplementary Table 2). All qPCR assays were done in triplicate using qPCRBIO SyGreen Blue Mix Lo-ROX (PCRBIOSYSTEMS) and the QuantStudio3 Real-Time PCR System (Themo Fisher). For the standard curve, cDNA from each sample of the groups/sections was pooled at equal concentrations. The analysis of fold change expression was done by the delta-delta Ct method with Wilcoxon and *t*-testing for significance.

### “Omic” integration and phenotypic association

Metabolites present in the liver of each animal were analyzed as described elsewhere (28). In summary, approximately 250 mg of frozen liver samples were homogenized in 10 mM phosphate buffer and prepared for nuclear magnetic resonance (NMR) spectroscopy. H-NMR analysis was run on a Bruker Avance 600 MHz spectrometer (Bruker, Billerica) and metabolite concentration was calculated with Chenomx Profiler (v. 8.1, Chenomx, Edmonton, AB). The resulting metabolite concentrations (?Mol/g of tissue) were then integrated with the transcriptome and microbiota results using the R package mixOmics (v 6.8.0) (29). For the integration of multiple datasets, microbiota relative abundance in intestinal contents as determined by 16S rRNA amplicon sequencing (11), and intestinal gene expression (for individual intestinal sections) combined with the liver metabolites, we used the Data Integration Analysis for Biomarker discovery using latent cOmponents (DIABLO) (30), from the mixOmics package, to identify highly correlated variables across all datasets that segregate between experimental groups. The pathway analysis was done with metabolites and genes selected as having high correlations with the other datasets using the Integrated Molecular Pathway Level Analysis (IMPaLA) (31).

Clustering of co-expressed genes was estimated using the Weighted Correlation Network Analysis (WGCNA) (v.1.68) (32), which was also used to calculate the correlation between modules (sets of co-expressed genes) with phenotypic data of interest, such as traits that were observed to change during malnourishment (11). This analysis was done using samples from all intestinal sections for the FF and Mal groups, and for comparison, modules of co-expressed genes were also calculated using samples from all intestinal sections of only the Mal group. The traits used in this study were as follows: body weight at the end of the fifth week of the experiment (kg, Final_Weight), bacterial translocation to the mesenteric lymph nodes (MLN—number of colony-forming units on sheep blood agar plates containing homogenized MLN collected at necropsy), crypt depth (mean_his_crypt), villi height (mean_hist_height), lamina propria thickness (mean_hist_LP), villi width (mean_hist_width), small intestine weight (kg, Small_Weight), small intestine weight (kg) -to- body weight (kg) ratio (Small_body_ratio), relative abundance of *Bacteroidetes* in intestinal contents (Bacteroidetes), relative abundance of *Firmicutes* in intestinal contents (Firmicutes), Bacteroidetes-to-Firmicutes ratio (bac_firm_ratio), z-score (z_score_animal), electrical conductance across jejunum mucosa (Conductance), paracellular permeability in jejunum mucosa (Paracellular_perm), and transcellular permeability in jejunum mucosa (Transcellular_perm). The histological traits were measured in 5-well oriented crypt-villi per sample on slides stained with hematoxylin/eosin. The electric conductance and mucosa permeability were analyzed *ex vivo*, using Ussing chambers to measure the flux of FITC-4000 to assess paracellular transport and flux of horseradish peroxidase, to assess transcellular transport (permeability) (11). The correspondence between modules was considered significant with a *p*-value of < 0.0001, which gives -log_10_ *p*-value > 4. For the correlations between modules and traits the significance was *p*-value of < 0.05.

## Results

### Influence of malnourishment on gene expression in the proximal small intestine

**Figure 1:**
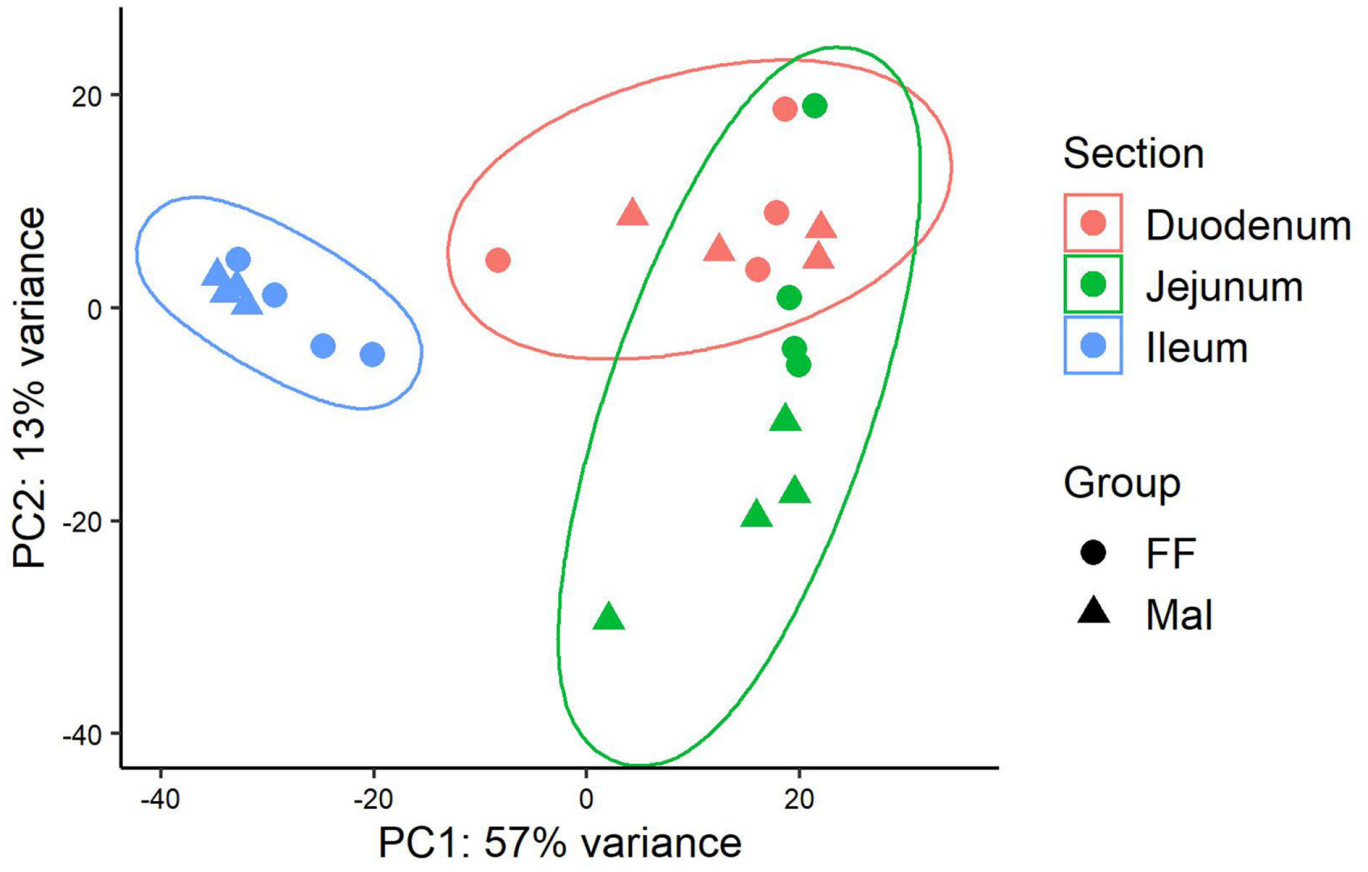
Principal component analysis of gene expression along the length of the intestinal tract in full-fed and malnourished pigs. Principal component analysis of the rlog transformed reads from Salmon for the full-fed (FF, *n* = 4) and malnourished (Mal, *n* = 4) groups in the duodenum (pink), jejunum (green), and ileum (blue).

**Figure 2:**
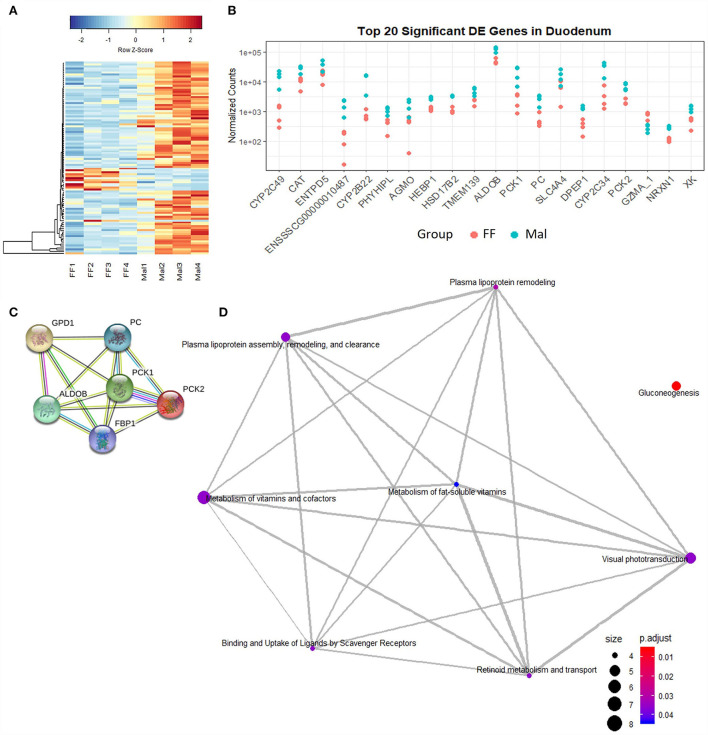
Gene expression and pathway analysis in the duodenum of full-fed and malnourished pigs. **(A)** Heatmap with hierarchical supervised clustering of the normalized counts using Euclidean distance for the 100 DE genes with the greatest variance between the full-fed (FF, *n* = 4) and malnourished (Mal, *n* = 4) groups in the duodenum. Z-score represents upregulated (red) and downregulated (blue) genes, with a color gradient indicating expression level. **(B)** Plot of the normalized counts of the 20 genes with the lowest padj values in the duodenum of FF [*n* = 4 (pink)] and Mal [*n* = 4 (blue)] animals. The genes are ordered left to right from lowest to greatest padj values. **(C)** Pathways significantly enriched in the duodenum when comparing the FF and Mal groups using GO and STRING functional analysis of upregulated genes associated with gluconeogenesis. Edge colors represent connection source: text mining connection (yellow), co-expression (black), curated databases (light blue), and experimentally determined (pink). **(D)** Reactome pathway analysis using the DE in the duodenum entrez gene ID in humans. Edges connect associated pathways. The size and color of nodes represent the number of genes up or downregulated and padj values, respectively.

Changes observed in the transcription profile were more evident along the length of the small intestine than between groups with different nutritional statuses (Figure 1). In the duodenum, a total of 173 genes were DE when comparing samples of FF animals and Mal animals (Supplementary Table 3). Of those, 145 genes were upregulated and 28 were downregulated in the Mal group (Figure 2A). The known genes that were most significantly different were cytochrome P450, family 2, subfamily C member 49 (CYP2C49), and catalase (CAT) (padj = 2.55 x 10-44 and 4.9 x 10-37, respectively), both upregulated in the Mal group, one associated with lipid metabolism and the other with oxidative stress (Figure 2B). Of the downregulated genes, the gene that was most significantly repressed was Granzyme A (GZMA) (padj = 3.2 x 10-13).

Gene ontology analysis (DAVID) showed that the only pathway significantly enriched in DE genes was gluconeogenesis (*p*-value = 0.00018). Genes including phosphoenolpyruvate carboxykinase (PCK) 1 and 2, guanosine-diphosphatase Gda 1 (GDP1), pyruvate carboxylase (PC), fructose-biphosphatase 1 (FBP1), and aldolase fructose-biphosphatase B (ALDOB) were upregulated in Mal animals (Figure 2C). Four of these genes (PC, PCK1, PCK2, and ALDOB) were among the DE genes with the 20 lowest padj values (Figure 2B). Using Reactome pathway analysis, in addition to the gluconeogenesis pathway (genes: PCK1, PCK2, FBP1, ALDOB, PC), metabolism of lipoproteins and associated pathways (genes: lipoprotein lipase (LPL), angiopoietin-like 4 (ANGPTL4), apoliprotein B (APOB), apoliprotein A1 (APOA1), scavenger receptor class B member 1 (SCARB1)) were also significantly enriched (padj < 0.5, Figure 2D).

Pathway analysis of upregulated genes in the duodenum between the FF and Mal groups named only gluconeogenesis and lipoprotein-associated pathways as being enriched with no presence of CYPs in any of them. However, once the human annotation of the DE genes was used, the most represented pathway in the GO analysis becomes the oxidation-reduction process (genes: AGMO, BBOX1, BCO1, CYP27A1, CYP2S1, CYP2W1, FA2H, HPGD, HSD17B2, HSD17B6, ME1, NDUFA4L2, NOS2, RDH16, SCD, SDR16C5, and UGDH; *p*-value = 1.11e−05) with gluconeogenesis having the second lowest *p*-value.

### Influence of malnourishment on gene expression in the mid-small intestine

**Figure 3:**
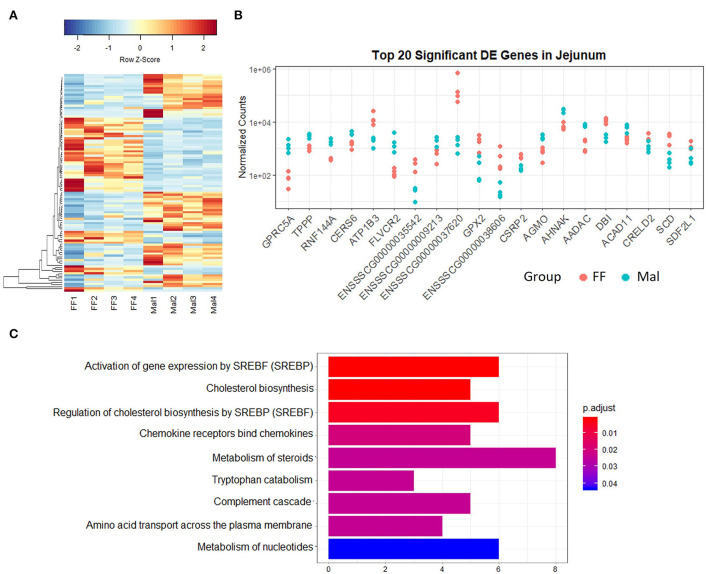
Gene expression and pathway analysis in the jejunum of full-fed and malnourished pigs. **(A)** Heatmap with hierarchical supervised clustering of the normalized counts using Euclidean distance for the 100 DE genes with the greatest variance between the full-fed (FF, *n* = 4) and malnourished (Mal, *n* = 4) groups in the jejunum. Z-score represents upregulated (red) and downregulated (blue) genes, with a color gradient indicating expression level. **(B)** Plot of the normalized counts of the 20 genes with the lowest padj values in the jejunum of FF [*n* = 4 (pink)] and Mal [*n* = 4 (blue)] animals. The genes are ordered left to right from lowest to greatest padj values. **(C)** Reactome analysis of downregulated genes in the jejunum of FF compared to Mal piglets in the jejunum. Bar length represents the counts of genes DE in each pathway, and colors represent the padj value.

The separation between the FF and Mal groups is more evident in the jejunum than in the other sections of the small intestine (Figure 1). This is corroborated by a greater number of DE genes (580), of which 361 were upregulated and 219 were repressed (Figure 3A). The top DE genes count plots and table with their log_2_ fold change and associated padj values are presented in Figure 3B and Supplementary Table 4, respectively.

The pathways with lowest *p*-values were response to toxic substance (*p*-value = 0.0005) and positive regulation of leukocyte chemotaxis (*p*-value = 0.003). All the DE genes associated with response to the toxic substance were membrane proteins with the majority associated with transmembrane transport [Aquaporin-10 (AQP10), solute carrier family 6 members 14 and 4 (SLC6A14, SLC6A4), transient receptor potential ion channel subfamily M member 6 (TRPM6), and gap junction protein (GJC2)]. Reactome analysis of the repressed genes (Figure 3C) displayed pathways associated with cholesterol biosynthesis. The pathway analysis of the upregulated genes revealed pathways associated with SLC-mediated transmembrane transport and transport of small molecules (data not shown).

### Influence of malnourishment on gene expression in the distal small intestine

**Figure 4:**
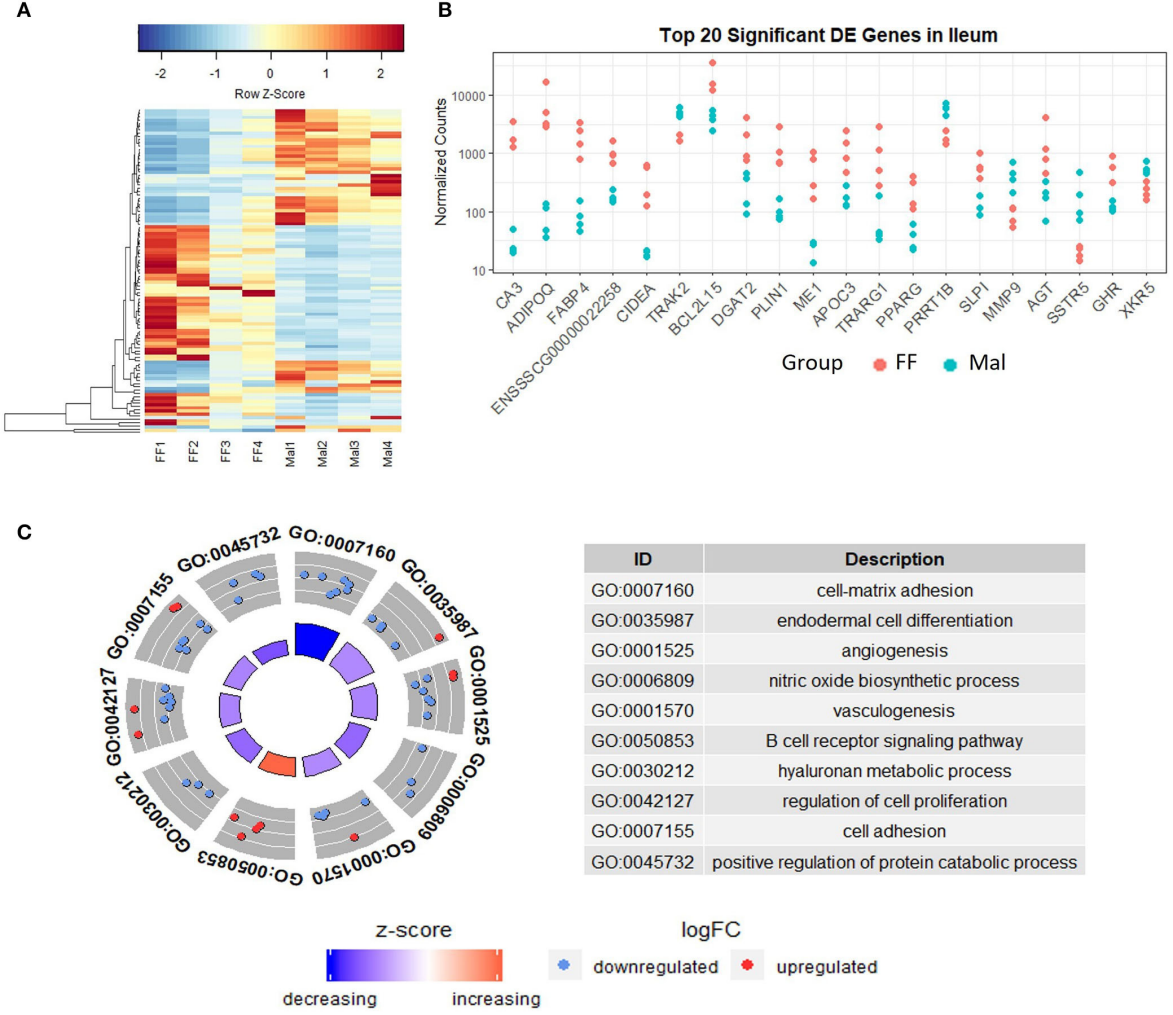
Gene expression and pathway analysis in the ileum of full-fed and malnourished pigs. **(A)** Heatmap with hierarchical supervised clustering of the normalized counts using Euclidean distance for the 100 DE genes with the greatest variance between the full-fed (FF, *n* = 4) and malnourished (Mal, *n* = 4) groups in the ileum. Z-score represents upregulated (red) and downregulated (blue) genes, with a color gradient indicating expression level. **(B)** Plot of the normalized counts of the 20 genes with the lowest padj values in the jejunum of FF [*n* = 4 (pink)] and Mal [*n* = 4 (blue)] animals. The genes are ordered left to right from lowest to greatest padj values. **(C)** Gene ontology analysis comparing the DE genes in the ileum of FF and Mal animals. The pathways displayed had *p*-values < 0.005, with the height of the inner circle of the graph representing significance and the color representing the pathway status (increased or decreased expression compared to FF samples). The outer circle represents the genes in the pathway that are up or downregulated in the malnourished group.

Gene expression in the ileum was distinct from that in the duodenum and jejunum (Figure 1) but despite similarities between Mal and FF expression profiles in the ileum, 475 genes were DE, 199 were upregulated, and 276 were repressed (Figure 4A). Among the top 20 DE genes according to the padj value, several genes associated with lipid metabolism and transport such as fatty acid binding protein 4 (FABP4), adiponectin (ADIPOQ), cell death inducing DFFA-like effector A (CIDEA), diacylglycerol O-acyltransferase 2 (DGAT2), perilipin 1 (PLIN1), and apolipoprotein C3 (APOC3) were all downregulated in Mal animals (Figure 4B, Supplementary Table 5). The gene with lowest padj value was carbonic anhydrase 3 (CA3) (log_2_FC = −5.9).

The GO and Reactome analysis agreed on the enrichment of the extracellular matrix organization and adhesion pathways for downregulated genes (Figure 4C and Supplementary Figure 1A). The GO analysis indicated an enrichment of cell-matrix adhesion (*p* = 0.0001) while the Reactome analysis indicated multiple associated pathways, including extracellular matrix organization (padj = 0.0005) and integrin cell surface interactions (padj = 0.0005), which were influenced mainly by the repressed genes. Immune pathways and gluconeogenesis were enriched in the upregulated genes (Supplementary Figure 1B). The absolute gene set enrichment score was greater for lipid metabolism and metabolism, influenced mainly by the genes with the lowest padj values such as CIDEA, PLIN1, FABP4, ME1, PPARG, AGT, and DGAT2.

### Effects of nutritional status common to all intestinal sections

**Figure 5:**
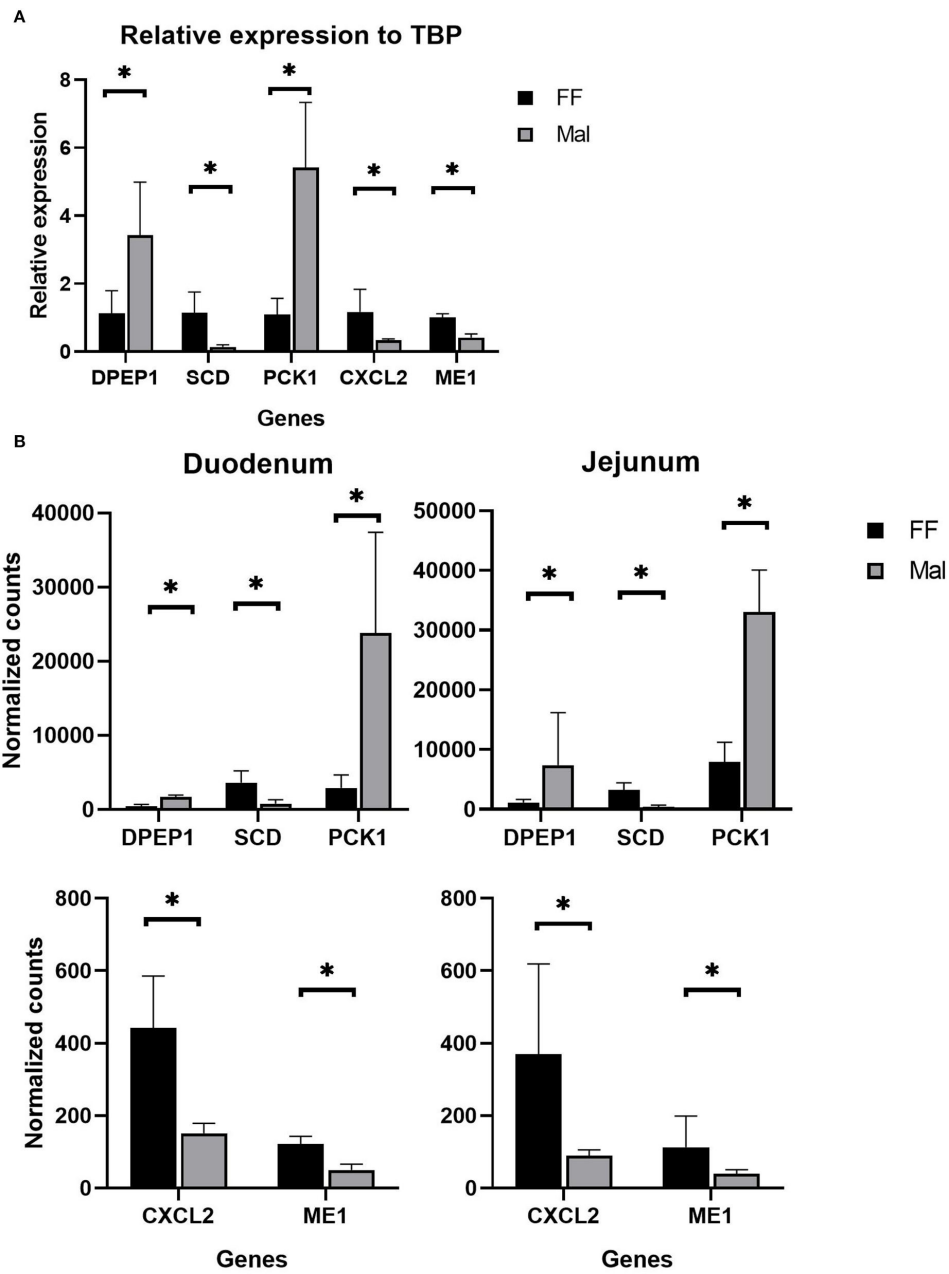
Validation of RNAseq data. **(A)** qPCR results of relative expression normalized to TATA box binding protein using jejunum (DPEP1, SCD, and PCK1) and duodenal samples (CXCL2 and ME1). *Significantly different (*p* < 0.05) by the Wilcoxon test. **(B)** Normalized counts from Salmon of animals from the full-fed (FF, *n* =4) and malnourished (Mal, *n* = 4) groups in the duodenum and jejunum. *Significantly different (*p* < 0.05).

Considering all pairwise comparisons made, only 25 DE genes were common to all segments of the small intestine (Table 1). From those, DPEP1, ME1, CXCL2, PCK1, and SCD were used to validate the RNA-seq results through qRTPCR (Figure 5). For DPEP1, PCK1 and SCD jejunal samples were used to test gene expression, while the remaining were tested on duodenal samples.

**Table 1:** DE genes in common to all small intestinal sections between the FF and Mal groups.

**Ensembl gene ID**	**Log_2_FC Duod**.	**Log_2_FC** **Jej**.	**Log_2_FC Ileum**	**Gene name**
ENSSSCG00000035347	3.06	1.88	1.11	ENSSSCG00000035347
ENSSSCG00000031367	2.44.58	2.54	1.1	ENSSSCG00000031367
ENSSSCG00000009213	1.3	−2.94	1.42	ENSSSCG00000009213
ENSSSCG00000007978	−2.59	2.27	−3.12	ENSSSCG00000007978
ENSSSCG00000011629	1.58	1.59	1.02	ACAD11
ENSSSCG00000015356	2.1	2.06	2.07	AGMO
ENSSSCG00000038300	1.34	1.24	1.39	ALDOB
ENSSSCG00000017861	1.13	1.76	1.86	ASPA
ENSSSCG00000008959	−1.53	−1.76	−1.77	CXCL2*
ENSSSCG00000023320	1.83	1.81	1.34	CYP3A39
ENSSSCG00000021971	1.95	1.86	1.21	DPEP1*
ENSSSCG00000004017	1.70	1.38	1.65	FRMD1
ENSSSCG00000027865	1.07	1.46	1	GALC
ENSSSCG00000029592	3.29	3.88	1.78	GPRC5A
ENSSSCG00000014725	−2.48	−2.96	−2.9	HBB
ENSSSCG00000038221	1.54	1.36	1.62	HSD17B2
ENSSSCG00000004454	−1.24	−1.42	−3.96	ME1*
ENSSSCG00000007436	1.91	1.21	2.37	MMP9
ENSSSCG00000017755	−1.99	−1.58	−1.83	NOS2
ENSSSCG00000007507	2.98	2.07	1.76	PCK1*
ENSSSCG00000002009	1.69	1.04	1.3	PCK2
ENSSSCG00000025969	1.71	1.7	1.54	PTPRR
ENSSSCG00000010554	−2.29	−2.93	−2.16	SCD*
ENSSSCG00000002032	1.94	2.47	1.2	SLC7A8
ENSSSCG00000030388	−1.41	−1.3	−1.14	UPP1

DESeq2 results from Salmon transcript abundance of the DE genes in all sections of the small intestine in alphabetical order. Log_2_FC– log_2_ fold change in abundance of the Mal group compared to FF. Duod, Duodenum; Jej, Jejunum. ^*^Genes used for qRTPCR for validation of RNAseq results.

The genes DPEP1 and PCK1 were significantly upregulated (*p* = 0.02 (Wilcox); *p*=0.002, *p* = 0.04 (t.test), respectively) in Mal, with a relative expression compared to the housekeeping gene TBP of 5.13 and 5.3 and fold change of 4.1 and 4.6, respectively (Figure 5A). The genes SCD, CXCL2, and ME1 were significantly (*p* = 0.02 (Wilcox); *p* = 0.004, *p* = 0.05, *p* = 0.002 (t.test), respectively) repressed (fold change of 0.1, 0.42 and 0.48, respectively) in the qPCR experiments (Figure 5A). These results corroborated the high-throughput results from the mRNA sequencing (Figure 5B). In the sequencing, the log_2_ fold change of DPEP1, PCK1, and SCD in jejunum samples was 1.86, 2.07, and−2.9, respectively, while CXCL2 and ME1 had log_2_ fold changes of −1.53 and −1.24, respectively, in the duodenum (Figure 5B).

### Gene co-expression analysis in the small intestine

Gene co-expression analysis was done using the samples from all three intestinal sections. The dendrogram representing the Euclidean unsupervised hierarchical clustering of the samples (Supplementary Figure 2) was similar to the sample PCA in Figure 1, with the samples of duodenum and jejunum intertwined and ileal samples agglutinated together. However, most of the phenotypic data diverged between animals with different diets, emphasizing animal weight and small intestine weight, both higher in the FF group, and paracellular permeability and conductance in the jejunum, lower in the FF animals (Supplementary Figure 2).

**Figure 6:**
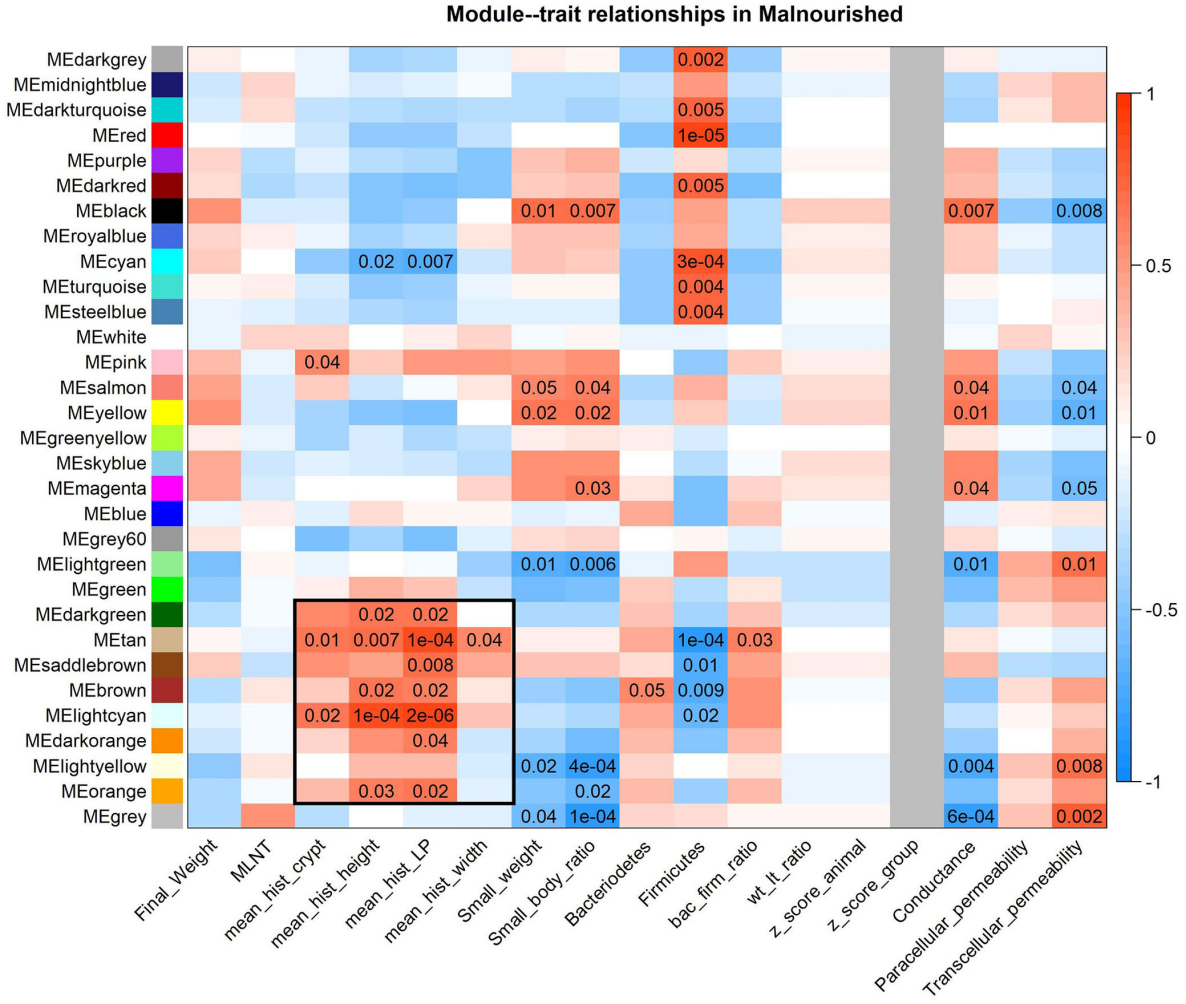
Heatmap of consensus modules-malnourished traits relationships. Consensus modules (clusters of co-expressed genes) made with samples from both the FF and Mal groups are identified as colors on the Y-axis and traits from Mal animals are on the X-axis. *P*-values of < 0.05 are displayed in the heatmap, color indicative of positive (red) or negative (blue) correlation between gene module and phenotype. The block of modules associated with the histological data is highlighted with a black box.

A block of consensus gene modules made with samples from the FF and Mal groups was significantly positively associated with histological features of malnourished animals and is visible in the module-traits relationship heatmap (Figure 6). More interestingly, this block of association is partially preserved when the phenotypic data from both groups are accounted for (Supplementary Figure 3), demonstrating that the association between these modules and the histological data agrees in both groups. Among these significant associations (Supplementary Table 6), there was a positive association between the tan module (368 genes) and villi height (*p*-value = 0.036) and lamina propria thickness (*p*-value = 0.0029).

The tan module had 74 (20%) genes that were DE in at least one intestinal section. Among them, coagulation factor 12 (F12), otopetrin 3 (OTOP3), uncoupling protein 3 (UCP3), all DE in the duodenum, C-C motif chemokine ligand 28 (CCL28), complement component 4 binding protein beta (C4BPB), and phospholipase A2 group III (PLA2G3) were DE in the jejunum and ileum. Most of the DE genes (59) were DE exclusively in the jejunum, with 17.9% of the genes in the module having different levels in the jejunum due to diet, including endoplasmin (HSP90B1).

The GO analysis of the tan module revealed 16 biological processes with *p*-values of < 0.05 (Table 2), many associated with oxidative stress and homeostasis (e.g., response to superoxide and oxidation–reduction process) and others associated with protein catabolism and degradation (e.g., tyrosine catabolic process, ER-associated ubiquitin-dependent protein catabolic process, and glycoprotein ERAD pathway), though none were considered significantly enriched (*p* < 0.0001). It is interesting to note that the negative correlation of the apoptotic process was also highlighted. The biological process of peripheral nervous system myelin maintenance was enriched in the DE genes in the module (data not shown). From the genes in this pathway, lanosterol synthase (LSS) and CYP51A1 were DE between groups in jejunum samples. The reactome analysis showed only one enriched pathway in cholesterol biosynthesis (padj = 0.03).

**Table 2:** Gene ontology analysis of the genes in the tan consensus module.

**Term**	**Description**	**Counts**	***P*–value**	**Genes**
GO:0030433	ER–associated ubiquitin–dependent protein catabolic process	7	2.38e−05	HSP90B1, UBXN4, EDEM3, ERLEC1, STT3B, EDEM2, PSMC6
GO:0032287	peripheral nervous system myelin maintenance	3	2.46e−03	SOD1, SH3TC2, NDRG1
GO:0006465	signal peptide processing	3	1.51e−02	SEC11C, IMMP1L, SPCS3
GO:0055114	oxidation–reduction process	7	2.27e−02	HPD, CYP51A1, PGD, SOD1, GGA2, MSMO1, DLD
GO:0090277	positive regulation of peptide hormone secretion	2	3.17e−02	TFR2, ILDR1
GO:0043686	co–translational protein modification	2	3.17e−02	STT3B, STT3A
GO:0006424	glutamyl–tRNA aminoacylation	2	3.17e−02	EARS2, EPRS
GO:0006572	tyrosine catabolic process	2	3.17e−02	HPD, TAT
GO:0043066	negative regulation of apoptotic process	8	3.83e−02	FAIM, DAB2, HSP90B1, HSPD1, MYC, PIM1, STAT5B, PTK2B
GO:0045454	cell redox homeostasis	4	4.12e−02	PDIA3, PDRX4, PDIA4, DLD
GO:0007229	integrin–mediated signaling pathway	4	4.57e−02	FGR, LAT, PTK2B, ITGB6
GO:0043687	post–translational protein modification	2	4.72e−02	STT3B, STT3A
GO:0000303	response to superoxide	2	4.72e−02	UCP3, SOD1
GO:0097466	glycoprotein ERAD pathway	2	4.72e−02	EDEM3, EDEM2
GO:1904382	mannose trimming involved in glycoprotein ERAD pathway	2	4.72e−02	EDEM3, EDEM2
GO:0072593	reactive oxygen species metabolic process	3	4.77e−02	PRDX4, SOD1, NDUFS1

Resulting from the comparison between consensus modules and phenotypic data from the malnourished group.

The consensus module light-cyan had the most significant module-trait relationship when only phenotypic data from Mal animals was considered (Supplementary Table 7), having a positive correlation with many histological features (Figure 6). The GO analysis of the genes in this module (285 genes, from those 34 were DE, from which 14 were DE exclusively in the jejunum, 5 in the duodenum, and 11 in the ileum) had 11 biological processes with a *p*-value of < 0.05, including proteolysis and one-carbon metabolic process (Table 3). The GO analysis for the DE genes in the module resulted in two highlighted pathways: response to retinoic acid (*p*-value = 0.02) and immune system (*p*-value = 0.04). The reactome analysis of the DE genes in the light-cyan module included pathways such as peptide ligand-binding receptors, cobalamin (Cbl, vitamin B12) transport and metabolism, and G(i) alpha signaling pathway, the latter being an inhibitory pathway of cAMP-dependent pathways (Supplementary Table 8). The reactome analysis for all the genes in the module indicated eight pathways as significantly enriched (Table 3).

**Table 3:** Pathway analysis of the genes in the light–cyan consensus module.

**Term**	**Description**	**Counts**	***P*–value/Padj**	**Genes**
GO:0015701	bicarbonate transport	3	0.0097	SLC26A7, SLC4A7, SLC4A4
GO:0007204	positive regulation of cytosolic calcium ion concentration	4	0.015	GLP1R, GPR33, CCR10, KNG1
GO:0006508	proteolysis	5	0.018	PROC, PGA5, TMPRSS15, CAPN6, Gastricsin–like
GO:0051453	regulation of intracellular pH	3	0.02	SLC26A7, SLC4A7, SLC4A4
GO:0006730	one–carbon metabolic process	3	0.022	AHCYL2, CA9, CA2
GO:0030501	positive regulation of bone mineralization	3	0.024	BMP7, BMP6, KL
GO:0046903	secretion	2	0.039	CA9, CA2
GO:0071281	cellular response to iron ion	2	0.039	TF, BMP6
GO:0015670	carbon dioxide transport	2	0.039	AQP5, CA2
GO:0030195	negative regulation of blood coagulation	2	0.039	PROC, KNG1
GO:0045665	negative regulation of neuron differentiation	3	0.041	BMP7, MIB1, ISL1
R–HSA−186712	Regulation of beta–cell development	7	0.00025 (padj)	NEUROG3, FOXA2, NKX2–2, PAX4, RFX6, NEUROD1, INSM1
R–HSA−140837	Intrinsic Pathway of Fibrin Clot Formation	5	0.001 (padj)	SERPINA5, F11, SERPINE2, KNG1, PROC
R–HSA−140877	Formation of Fibrin Clot (Clotting Cascade)	5	0.01 (padj)	SERPINA5, F11, SERPINE2, KNG1, PROC
R–HSA−210745	Regulation of gene expression in beta cells	4	0.01 (padj)	FOXA2, NKX2–2, NEUROD1, RFX6
R–HSA−425381	Bicarbonate transporters	3	0.01 (padj)	SLC4A7, AHCYL2, SLC4A4
R–HSA−8957275	Post–translational protein phosphorylation	7	0.02 (padj)	PENK, AFP, KNG1, TF, PROC, MIA3, ITIH2
R–HSA−381426	Regulation of Insulin–like Growth Factor (IGF) transport and uptake by Insulin–like Growth Factor Binding Proteins (IGFBPs)	7	0.04 (padj)	PENK, AFP, KNG1, TF, PROC, MIA3, ITIH2
R–HSA−425407	SLC–mediated transmembrane transport	10	0.04 (padj)	SLC28A3, SLC26A7, SLC11A1, SLC16A7, SLC6A11, SLC4A7, SLC27A6, AHCYL2, SLC4A4, SLC38A3

Resulting from the comparison between consensus modules and phenotypic data from the malnourished group.

### Omics integration

To uncover systemic fallouts from malnourishment, the transcriptome and microbiota (16S) data from the duodenum, jejunum, and ileum was integrated with liver metabolome data from the same animals. In all three intestinal sections, there was high similarity in the transcriptomic (DE genes between FF and Mal groups), metabolome, and microbiota (Supplementary Figure 4). Also, a difference between the FF and Mal animals was seen in all three omics datasets along the intestinal tract.

#### Duodenum

**Figure 7:**
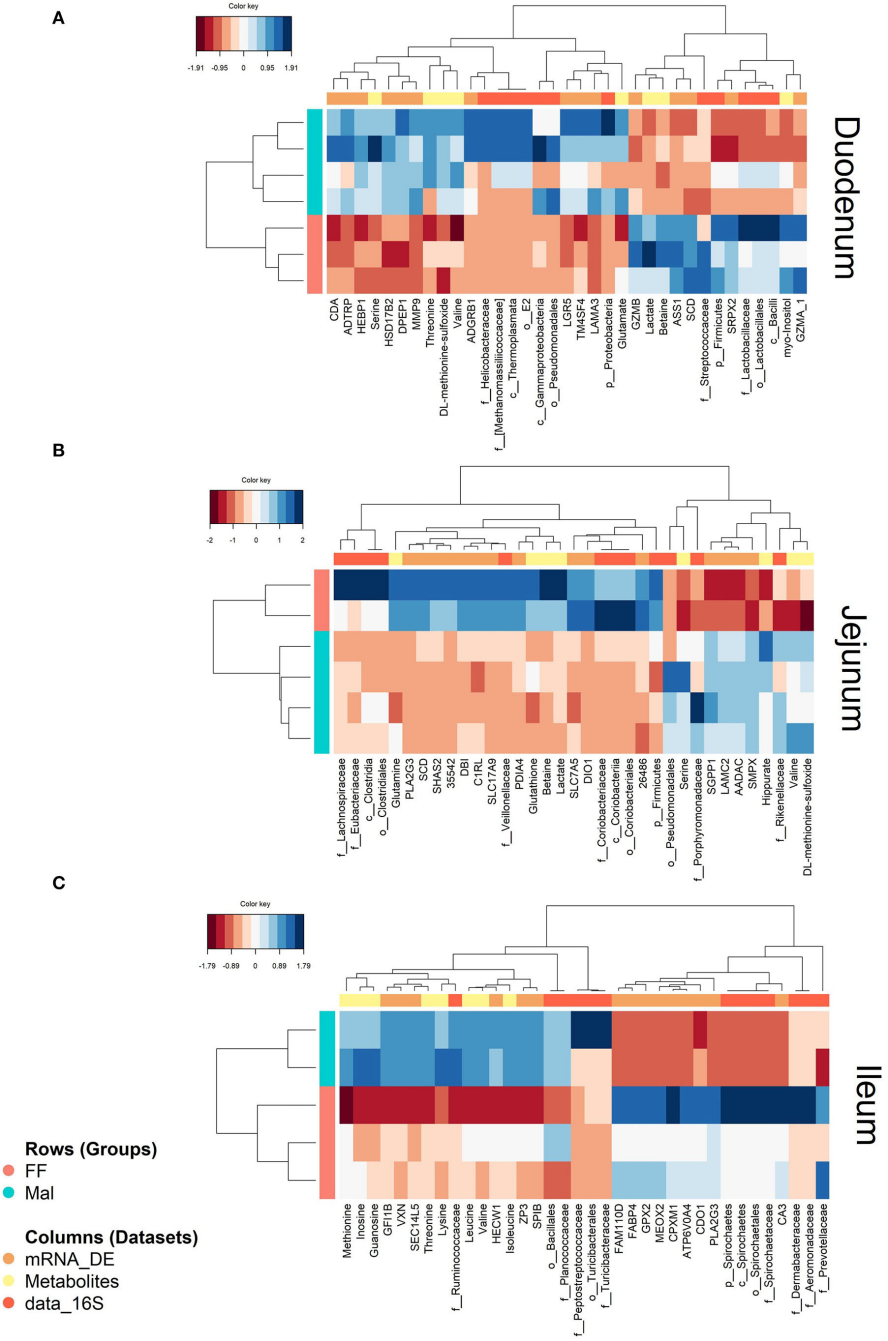
Heatmaps showing correlations between DE genes (mRNA, orange), microbiota (data_16S, yellow), and liver metabolites (metabolites, dark orange) in full-fed (FF, pink) and malnourished animals (Mal, blue) in the **(A)** duodenum, **(B)** jejunum and **(C)** ileum. The color key represents the level of the variable in the sample from negative (dark red) to positive (dark blue).

Correlation between variables from different datasets from the duodenum can be observed clearly in the heatmap (Figure 7A), where two blocks of correlated variables exist between the groups. One block contains, among others, the metabolites serine and threonine, *Proteobacteria* and several genes, all decreased in the FF group. The other block contains betaine, lactate, and myo-inositol, *Firmicutes* and genes, including SCD, all decreased in the Mal group.

The pathway analysis combining the genes and the metabolites revealed as enriched amino acid metabolism (Wikipathways; genes: PCK1 and ASS1; metabolites: citrate, valine, urea, glutamate and threonine; joint *p*-value = 9.46e-07), gluconeogenesis (Reactome; genes: PCK1 and FBP1, metabolites: citrate and glutamate; joint *p*-value = 8.36e−05), metabolism (Reactome; genes: GPD1, DPEP1, DGAT2, SCD, PCK1, CDA, HSD17B2, FBP1, ASS1, ACOT12; metabolites: citrate, mannose, valine, glutamate, urea, betaine, myo-inositol, cytidine; joint *p*-value = 0.0003), and metabolism of lipids (with tendency for enrichment, q-value = 0.056; Reactome; genes: GPD, DPEP1, DGAT2, SCD, ACOT12, HSD17B2; metabolites: citrate, glutamate and myo-inositol; joint *p*-value = 0.003).

#### Jejunum

In the jejunum, the samples also mirrored each other in the different datasets where all three datasets were only available for six animals (two from the FF and four from the Mal group). As was seen in the duodenum, there was greater similarity between the metabolite and transcriptome data than between the metabolite and the 16S information, but all the correlations were > 0.98 (Supplementary Figures 4A,B).

The circle plot shows blocks of overlapping genes, metabolites, and OTUs, with one block containing only metabolites and genes (metabolites: hippurate, valine, DL-methionine-sulfoxide; genes: AADAC, SGPP1, LAMC2, SMPX, and SGPP1) and one block containing variables from all three datasets comprising the co-localization of betaine, DIO1, PLAG2G3 and *Coriobacteriaceae* (Supplementary Figure 5). Also, in this block, lactate and glutamine overlapped the genes DBI, SLC7A5, and SHAS2 and the OTUs *Coriobacteriaceae, Veillonellaceae*, and *Firmicutes*. In the heatmap (Figure 7B), two distinct blocks of related variables were again evident between the groups. One block related, among others, the metabolites glutathione, betaine, and lactate to *Veillonellaceae* and multiple genes, all diminished in the Mal group, and the other block showed the correlation between valine, DL-methionine-sulfoxide, *Rikenellaceae*, and multiple genes that were all more prevalent in the Mal group. The variables overlapping betaine and lactate are presented in Supplementary Table 9. The pathway analysis of the genes and metabolites highly associated with betaine and lactate resulted in no significant (joint *p*-value of >0.0001) enrichment.

#### Ileum

When combining the liver metabolites, the transcriptome and microbiota data from ileum samples, as in the previous sections there were high similarities between datasets. Samples from FF animals agglomerated together in all three datasets and greater variance in the Mal group can be observed in the correlation matrix plot comparing the datasets (Supplementary Figure 4C). In this plot, it is also seen that the metabolite and 16S data segregate for the two groups more than the transcriptome data, although, as in the other sections, metabolite and transcriptome data were closer than the 16S data, in this case, the latent component 1 was used to describe these datasets as identical.

The blocks with the highest correlation can be seen in the heatmap (Figure 7C). One of them is a conglomerate containing the genes CA3 and glutathione peroxidase 2 (GPX2) with the relative abundance of the family *Spirochaetaceae*, all increased in the FF group. The association between inosine, methionine, and guanosine and the taxonomic families *Ruminococcaceae* and *Planococcaaceae* can also be observed in another block, all elevated in the Mal group. The difference between groups was more subtle than in the proximal intestinal sections.

## Discussion

Malnourishment is a frequent occurrence in young children in developing countries that can have life-long consequences. Using a pig model of childhood malnutrition that recapitulates many of the disease markers in humans including impaired intestinal barrier function and structure as well as altered blood chemistry and microbial profiles (10), we observed changes in gene expression in the small intestine that were related to energetic balance, cellular proliferation, response to stress, and immune response. Due to the small number of samples, the manuscript has the primary goal to be a hypothesis-generator, identifying possible links between the phenotype and the omics data that should be further investigated in future studies.

### Energetic balance (gluconeogenesis and lipolysis)

Pathway analysis of DE genes in the duodenum highlighted the process of gluconeogenesis as being distinct between the FF and Mal groups. Indeed in the duodenum, four of the top 20 DE genes (*ALDOB, PCK1, PCK2*, and *PC*) were related to gluconeogenesis, and genes *FBP1* and *GDP1* were also DE between the groups, all genes being upregulated in the Mal animals. Gluconeogenesis is the process of production of glucose from non-carbohydrate substrates (e.g., fatty acids and proteins) mainly occurring in the liver with the objective of maintaining blood glucose levels. As blood glucose levels were significantly lower in Mal animals after both 3 and 5 weeks of the restricted diet (11), and it is known that intestinal gluconeogenesis is induced during fasting and diabetes (33, 34), this highlights the ability of intestinal tissue to compensate for low glucose levels, which was also demonstrated *in vitro* in low glucose conditions (35).

Increased gluconeogenesis in the Mal animals was corroborated by results obtained when integrating the multiple datasets where a correlation was found between hepatic lactate and betaine and the OTU *Lactobacillaceae* in the duodenum, all of which were decreased in Mal animals. Lactate is a by-product of glucose metabolism in anaerobic conditions and is mainly processed by the liver into pyruvate for gluconeogenesis (36). Hepatic lactate was significantly lower in Mal animals compared to FF controls (28) and suggests an increase in gluconeogenesis in hepatocytes, which is known to happen in low blood glucose level conditions (37). Lactate is also produced by *Lactobacillus* and its production has been shown to be enhanced by betaine due to an osmoprotectant function (38). Along with decreased levels of lactate, hepatic betaine levels tended to be decreased (28) which could be linked to the decrease in *Lactobacillu*s in duodenal intestinal content and feces of Mal animals and suggests osmodysregulation in the liver. The relationship between *Lactobacillus* and levels of these metabolites can be illustrated in studies with germ-free mice that produced the opposite effect of what was seen here. Germ-free mice supplemented with galacto-oligosaccharides plus *Lactobacillus rhamnosus* showed a relative increase of betaine in the liver (39) and those colonized by *Lactobacillus paracasei* (40) had an increase in lactate and decrease in serine in the intestines. Further supporting the role of *Lactobacillus* in our model, hepatic serine levels were positively correlated with several genes in the duodenum, and these genes were negatively correlated with *Lactobacillaceae, Firmicutes*, betaine, and lactate, reinforcing the crosstalk between microbiota, metabolites, and gene expression.

In the lower small intestine, energy-related processes besides gluconeogenesis were predominantly impacted by malnourishment with Mal animals having an increase in lipolysis and a decrease in lipogenesis. In the ileum, lipid and fatty acid metabolism was highly affected by many genes associated with these processes in the top 20 DE genes. Among the most DE genes in the ileum, ADIPOQ is mainly expressed by mature adipocytes and encodes adiponectin, a protein hormone involved in body weight regulation. The plasma level of adiponectin and ADIPOQ mRNA in adipose tissue is decreased in obese and diabetic conditions (41). Thus, the higher expression of ADIPOQ in FF animals might indicate greater adipocyte presence in the tissue of animals with no restricted diet. Likewise, FABP4, also upregulated in FF animals, is also expressed mainly by adipocytes but in contrast has increased levels associated with obesity and insulin resistance (42).

Another gene predominantly expressed in adipocytes was CIDEA, which is also expressed in the intestinal tract including the small intestine and colon (human protein atlas). Expression of this gene, which has been associated with lipolysis and obesity with CIDEA-null mice being resistant to diet-induced obesity (43), was downregulated in Mal animals. Interestingly, in human white adipose tissue, the expression of CIDEA decreased by 50% in obese subjects who also had 2-fold greater adipocyte lipolysis values than non-obese subjects (44). The downregulation of CIDEA could indicate a similar pattern with decreased expression associated with increased lipolysis in the ileum of Mal animals. In addition, the expression of CIDEA is inversely correlated to TNF-alpha secretion in preadipocytes (44), with TNF-alpha downregulating the transcription of CIDEA mRNA (45). Interestingly, a restricted diet in obese women increased the expression of CIDEA in adipose tissue (46) indicating that either the intestinal expression is regulated differently, or that nutritional status has a big impact on CIDEA regulation. If the tendency is the same in the small intestine, this suggests an inflammatory state in the distal small intestine as is corroborated with the enrichment of immune response pathways in upregulated genes in Mal animals. More interestingly, CIDEA not only inhibits lipolysis but also inhibits TNF-alpha secretion without changing its transcription level (44). We did not observe differences in expression of TNF-alpha between groups, but two TNF receptors (TNFRSF13C and TNFRSF9) were upregulated in the ileum of Mal animals (44) also demonstrated that a decrease in CIDEA caused a decrease in PLIN which was also observed in the ileum of Mal animals, with PLIN1 and PLIN4 being repressed. Perilipin is lipid droplet protein associated with the protection against lipases and consequently against lipolysis (47), helping explain the pathway enrichment for lipolysis in Mal animals.

Also associated with lipid metabolism, TRAK2 was upregulated in the ileum of animals kept on a restricted diet. This transcript encodes a protein involved with the regulation of organelle trafficking and was shown to regulate HDL plasma concentration and cholesterol efflux to apolipoprotein A-I by inhibiting the expression of ABCA1 in macrophages and liver cells (48). In the ileum of Mal animals, the transcript of ABCA6 was downregulated mimicking the relation between TRAK2/ABCA1 observed in the liver, while apolipoprotein C-3 (APOC3) was also downregulated in the Mal animals. The TRAK2 gene was in a module of co-expressed genes significantly inversely correlated with small intestine weight across both groups (black consensus module, data not shown). APOC3 is an apolipoprotein expressed in the liver and small intestine and inhibits the uptake of triglyceride-rich lipoproteins. Its absence has been shown to increase lipolysis of very-low-density lipoprotein (49, 50), indicating an increase in lipolysis in Mal animals, corroborating with the decrease of CIDEA/PLIN1 discussed previously.

Reduction in lipogenesis is also suggested by the decreased expression of genes involved in this process in Mal animals. The final step of triglyceride synthesis is carried out by DGAT enzymes and DGAT1 expression was decreased in Mal animals, suggesting lower efflux of lipoprotein from the intestine of Mal animals. Also supporting the reduction in lipogenesis is the downregulation of ME1 and FASN in Mal animals. ME1 is required for the formation of NADPH, which is necessary for fatty acid biosynthesis in the intestinal tract by fatty acid synthetase, encoded by the FASN gene. ME1 is also associated with diet-induced adiposity, and the knockout of ME1 in male mice caused significant decreases in FASN expression in the jejunum and a decrease in crypt depth in the colon (51). This agrees with our finding that Mal animals had consistently shallower crypts in all intestinal sections including the ileum (10, 11).

### Cell-growth, cell proliferation, and cell turn-over

Malnourished animals weighed half as much as their FF counterparts and had significant reductions in villi height, villi width, crypt depth, and lamina propria thickness in each section of the small intestine (11). Intestinal stem cells (ISCs) located in the villi crypts are responsible for the renovation of the different cells present in the mucosal layer through mitosis and differentiation. The downregulation of genes with mitotic function can cause the shortening of villi and fasting has been shown to decrease ISC cell proliferation in mice with consequent reductions of villus height (52) with increased cell proliferation having the opposite effect (53–55). Remarkably, the co-expression module that was positively associated with histological features such as villi height, and lamina propria thickness had no genes associated with mitotic function but was enriched for genes with proteolytic function and response to oxidative stress, indicating that proteolysis may be the energetic source for mitotic division and that an effective response to oxidative stress is also necessary for normal morphophysiological maintenance of the mucosa.

However, when we considered additional animals from a simultaneous study, the downregulation of several key mitotic factors was evident. These samples were derived from malnourished animals with dietary supplementation of 500 ml of regular goat milk or lysozyme-rich goat milk (11). As the effect of milk supplementation was subtle compared to the effect of nutritional status, for some analyses against the FF animals, we combined samples from all three malnourished groups independently of milk supplementation to increase the power and help reveal genes that maybe be masked by within-group variation. Several genes with mitotic function were repressed in the duodenum of malnourished animals independently of milk supplementation including *BUB1, BUB1A, KIF18A*, and *CENPA*, and many others including highly connected kinetochore proteins and cyclins, corroborating the halting of replication of epithelial cells.

Studies have shown that a drug that mimics caloric restriction (metformin) and decreases systemic insulin levels, also results in the downregulation of several key mitotic factors including *CENPA, SGO1*/*SGOL1*, and *BUB1* (56, 57), all of which were downregulated in the duodenum of malnourished-animals independent of milk supplementation. These mitotic factors are regulated by AMP-activated protein kinase (*AMPK*), a conserved low-energy checkpoint that inhibits cell proliferation upon sensing metabolic status. *AMPK* is activated when there are low cellular levels of ATP and high levels of AMP, such as during glucose deprivation and heat shock (58). In addition, insulin signaling has been implicated in the expression regulation of mitotic factors such as *CENPA* with growth factors guiding the proliferative state according to metabolic demand in pancreatic beta cells (59). Furthermore, the induction of ISC proliferation with a consequent increase in villus height and crypt density was observed in diet-induced obese mice, and this induction was significantly correlated to plasma insulin levels (55). Evidence that the cellular caloric restriction due to reduction in systemic insulin could hinder cell proliferation connects the two major effects of malnutrition, decreased blood glucose levels and stunting, both of which were observed in our pig model (11).

The ileum had a different regulation of cell proliferation with many insulin-independent pathways being altered in the repressed genes in the restricted diet group, including CA3, insulin-like growth factors binding proteins (IGFBP) 3, 5, 6, and 7, and growth hormone receptor (GHR). The decrease in CA3 in the Mal group may be related to a decrease in intestinal motility, since CA3 is increased during oxidative stress resulting from exercise and skeletal muscle damage (60). Furthermore, fasting is known to decrease gastrointestinal motility (61). The lower CA3 expression could also indicate a breach in the intestinal defense, since CA3 downregulation was correlated to hepatitis viral infection and progression (62). Metagenomics of Mal animals also indicated a higher viral abundance (Chew S. et al., data not published).

Other genes associated with smooth muscle contraction (ACTA2, DYSF) and cell proliferation (IGFBP3 and IGFBP5) were also downregulated in Mal animals. While IGFBP-3 and 5 regulate cell proliferation and cell growth by modulating the availability of the insulin-like growth factor I (IGF-I) to insulin-like growth factor I receptor (IGF-R), both IGFBPs are produced by intestinal smooth muscle cells acting as autocrine regulators of cell proliferation (63, 64). In addition, IGFBP-5 can stimulate growth in intestinal smooth muscle cells (65) independently of IGF-I/IGF-IR with high expression of IGFBP-5 being observed in rapidly dividing intestinal smooth muscle cells, and its expression progressively decreasing with declining cell growth rates (63), again potentially indicating a decrease in intestinal motility.

IGF-I is predominately produced by the liver, and although it can be expressed locally in the intestine, its expression level has been shown to not change in the ileum during intestinal adaptation studies with massive small bowel resection (66). The proliferative effects of systemic growth hormone (GH) are in part mediated *via* IGF-1 through the GHR (67). While fasting decreases serum IGF-I concentrations and consequently increases GH secretion (68), the downregulation of GHR in the ileum of Mal animals could counterbalance the increase in GH, further corroborating the decrease in cell proliferation and growth due to the restricted diet. However, the gene co-expression analysis indicated the presence of GHR in modules significantly and inversely correlated with lamina propria thickness and villi height (data not shown brown module, Mal exclusive, and consensus). The immunoreactivity of IGF-I and IGFBP3 was increased in the small intestine of rats after GH injections (69). These increases were accompanied by an increase in villi height, crypt cell number, and epithelium thickness. These results agree with the micromorphology observed in the ileum of Mal animals, with a significant decrease in villi height, lamina propria thickness, and crypt depth and could indicate that the decrease in IGFBP-3 might be associated with the decrease in GHR expression.

In the ileum of Mal animals, 13 different collagen genes, including two chains of collagen type I, were repressed. The dysregulation of collagen and other extracellular matrix proteins could be connected to the lower expression of IGFBP-3 and 5. IGF-I increases both expression of collagen type I alpha I (COL1A1) and IGFBP-5 (70, 71) with IGFBP-5 enhancing IGF-I mitogenic activity in fibroblasts by associating with extracellular matrix and acting as a modulator of IGF-I activity and protecting it against degradation (72–74). IGFBP-3 has also been shown to increase the expression of collagen type I in the intestine (75). Overall, multiple pathways were implicated in the stunted growth of the intestinal mucosa of Mal animals and offered potential targets for nutritional-based therapeutics.

### Response to stress

Cytochrome P450 proteins (CYPs) are enzymes crucial for metabolic and biosynthetic function, but that generate reactive oxygen species (ROS) during their reaction cycles. ROS are detrimental to cellular structures leading to damage and cell death (76, 77). The upregulation of *CYP2C49* in the duodenal samples of Mal animals is interesting since its gene expression in the jejunum is associated with lipid metabolism, and it has also been shown to be associated with body-fat deposition and composition in weaned piglets (78). Its expression is also inversely associated with the administration of a probiotic of the *Lactobacillus* genus, which has been shown to have a lower relative abundance in the feces of the Mal animals (11). Interestingly, a diet with high content of saturated fat also increased the expression of this gene, as well as the gene *CYP3B22* (79) was also upregulated in the duodenum of the Mal animals.

Five other CYPs (*CYP3A39, CYP2W1, CYP27A1, CYP2S1*, and *CYP2C34*) were upregulated in the duodenum, indicating that the regulation of the cytochrome 450 systems may have an important impact on the response to a restricted diet. Furthermore, this occurred not only in the duodenum, but also in the jejunum, which had upregulation of six (*CYP3A39, CYP2W1, CYP2C49, CYP4F2, CYP2C34*, and *CYP2B22*) of the CYPs DE in the duodenum. In addition, the jejunum had a CYP that was downregulated in Mal animals (*CYP51A1*) that has been shown to be upregulated in the intestines of fish fed with a high carbohydrate diet (80), indicating differential responses to diet-induced CPY expression.

In the jejunum, 28 of the 580 DE genes were associated with oxidation-redox processes, including *CYP2W1, CYP51A1*, and stearoyl CoA desaturase (SCD), with *SCD* repressed in all three intestinal sections. Nine other genes (upregulated: *ABCC2, VNN1, STC2, DUSP1, GAB1*, and *SELENOP*; downregulated: *APOE, ANGPTL7*, and *GPX2*) were also associated with response to oxidative stress. *ABCC2, VNN1*, and *APOE* were also DE in the duodenum together with *DUSP18, GPRC5A*, and *ANGPTL4*. Vanin-1 (VNN1) induces oxidative stress in colon cancer cells, and it is stimulated by the expression of G protein-coupled receptor class C group 5 member A (GPRC5A) (81). Both *VNN1* and *GPRC5A* were upregulated in the Mal group when compared with FF animals, again pointing to oxidative stress in the intestine.

The most representative pathway for DE genes in the duodenum was the oxidation-reduction process in agreement with the findings of the liver metabolomic data from the same animals where glutathione levels were 70% lower in the Mal group compared to FF animals (28), consistent with observations in rat models of malnutrition (82, 83). Furthermore, glutathione peroxidase 2 (GPX2), the intestinal-specific GPx enzyme responsible for reducing hydroperoxides by oxidizing glutathione (84) was downregulated in the jejunum of Mal animals compared to FF animals. The depression of GPx genes has also been observed in malnourished children (85).

Glutathione is an important antioxidant responsible for maintaining oxidative status and detoxification. Interestingly, ATP-binding cassette (ABC) transporter proteins, multidrug resistance protein 2 (ABCC2/MRP2), work as an antioxidant by removing toxic chemicals conjugated to glutathione out of the cells (86–90), and a glutathione S-transferase (GSTM3) was also upregulated in the duodenum of Mal animals. MRP2 is mainly expressed in the apical membrane of enterocytes, pumping glutathione-oxidant conjugates into the intestinal lumen and reducing their absorption (86, 90, 91). In the jejunum, hepatic glutathione was positively associated with the expression of *SCD* and the bacterial family *Coriobacteriaceae*, whose abundance has been associated with altered metabolic parameters including hepatic detoxification pathways (92), all of which were lower in Mal animals compared to FF. Even more interestingly, the module of co-expressed genes positively associated with villi height and lamina propria thickness showed enrichment of genes associated with the oxidation-reduction process, response to superoxide and cell redox homeostasis, including *CYP51A1*.

Some of these genes, such as GPX2, SCD, and GPRC5A, followed similar trends in the distal small intestine indicating oxidation-redox disbalance along the entire length of the small intestine. Furthermore, the decrease in CA3 in the ileum of the Mal group corroborates the potential vulnerability to reactive oxygen species. This enzyme has been shown to protect muscle, fibroblast, and liver cells against oxidative stress (60, 93–95) by hindering the apoptotic process and increasing cell growth after exposure to H_2_O_2_ (93) with its decrease suppressing the defense against free radicals (94). The genes CA3 and GPX2 were positively correlated with *Spirochaetes* and inversely correlated with *Planococcaceae*. There is evidence of some *Spirochaetaceae* lacking genes for oxidative stress response (96) and of correlations between the fecal abundance of this phylum and host genes associated with oxidative stress response such as NQO1 (97), which was also downregulated in the ileum of Mal animals. Although the difference in *Spirochaetaceae* abundance was not significantly different in the ileum, the fecal samples from the group on the restricted diet were enriched in *Spirochaetaceae* (11).

### Immune response

Immune response pathways, mainly associated with B-cell function and humoral response, were enriched in the up-regulated genes in the lower small intestine of Mal animals. Among them was Bruton tyrosine kinase (BTK) which is essential for mature B-cell development but is also present in a variety of immune cells such as macrophages (98). Mature and immature B-lymphocytes express BLK proto-oncogene, Src family tyrosine kinase (BLK), hemopoietic cell kinase (HCK), and spleen-associated tyrosine kinase (SYK) (99), and these Src tyrosine kinases (SYK, BLK, and BTK) are activated rapidly after B-cell receptor binding (100). Combined with the upregulation of the B-cell antigen receptor molecule (beta chain—CD79B), this indicates a larger B-cell population or an increase in activation of the mature B-cell population in Mal animals. Kelch-like family member 6 (KLHL6) was also upregulated in the restricted diet group. It is not only involved in B-cell lymphocyte antigen receptor signaling, but also in germinal center B-cell maturation (101). BLK, LCK, and CD79B have been shown to be upregulated in the ileum of mice that were orally supplemented with two *Lactobacillus* strains (102). The phylum *Lactobacillales* was significantly less abundant in the feces of Mal animals, indicating that the upregulation in these genes was not related to the increased abundance of this phylum. All the genes cited above, plus the interleukin-4-induced gene 1 (IL4I1), were present in a module (both Mal specific and consensus module—turquoise) that was significantly positively correlated with *Firmicutes* abundance in intestinal sections (data not shown).

The gene *IL4I1* was repressed in Mal animals, and it codes an immunosuppressant that inhibits T-lymphocyte proliferation and activation (103, 104) and promotes regulatory T-cell differentiation (105). Also, among the downregulated genes in the ileum was the porcine gene alveolar macrophage-derived neutrophil chemotactic factor II (AMCF-II). The expression of this gene has been positively associated with the expression of IL17, which is mainly expressed by Th17 cells. IL4I1 has been shown to increase the differentiation of T-cells into Th17 in disfavor of Th1 and Th2 (105) indicating T-cell dysfunction in Mal animals.

The gene IL17-B and the receptor IL17REL were also downregulated in the distal small intestine of Mal animals, and while AMCF-II is a neutrophil chemoattractant, so are IL17-B (106) and CXCL2 which were also downregulated in the ileum of the restricted diet group. In the omic integration results, there was a positive correlation between AMCF-II, CXCL2, the phylum *Streptococcaceae*, and the hepatic gluthatione level. *Streptococcaceae* was less abundant in the feces of Mal animals (11) and CXCL2 was also present in a co-expression module that is significantly positively correlated to all histological features and inversely correlated to *Firmicutes* abundance (lightcyan, consensus module), while AMCF-II was in a module (Mal, yellow, consensus, black) negatively correlated with transcellular permeability. These findings indicate crosstalk between specific immune, histological, and microbial features in the lower small intestine.

Cell adhesion and cell matrix adhesion also seem to be dysregulated, with associated pathways being significantly enriched in the repressed genes in the ileum of Mal animals. The maintenance of the epithelial layer is important to intestinal homeostasis and inflammation control with many pathogen-recognition receptors being differently secreted in the luminal and basal face of the epithelial layer. Genes encoding proteins present in tight junctions that control the cell adhesion of the mucosal layer were mainly downregulated due to diet restriction. T-cadherin (CDH13) and cadherin (CDH5) were among the downregulated genes in the ileum of Mal animals, and as these genes encode important tight junction proteins, this indicates a possible increase in paracellular permeability in the epithelial layer of ileal intestinal mucosa as was seen in the jejunum (11). CDH13 was present in the darkgreen Mal-specific module that was significantly positively correlated with paracellular permeability corresponding with the increased transcellular and paracellular permeability observed in the jejunum of Mal animals (11). CDH13 genotype and expression have also been linked to an increased risk of *Campylobacter jejuni* infection (107), and the Campylobacter phylum had a 4-fold increased abundance in the feces of Mal animals, indicating an increased risk of infection.

## Conclusion

Alterations in oxidation-redox processes and depletion of antioxidants such as glutathione indicate an imbalance that could cause oxidative stress as demonstrated by pathway analysis of DE genes and hepatic metabolomic data from the animals. Furthermore, the downregulation of genes associated with ISC proliferative state and upregulation of gluconeogenesis genes due to restricted diet and the presence of metabolic sensors connecting cellular and systemic metabolic status with proliferation rate in intestinal cells indicate that prolonged reduction of caloric intake mimicked fasting in the Mal animals and caused a systemic decrease in blood glucose/insulin levels that induced intestinal gluconeogenesis along the length of the small intestine accompanied by lipolysis, especially in the ileum. The decrease in blood glucose and downregulation of growth hormone receptors were also linked to the decreased cell proliferation in the epithelial layers causing the shortening and thinning of villi structures.

These data suggest a connection between the morphophysiological conditions of malnourished animals with gut gene expression. It is important to understand the molecular effects of restricted nutrient and caloric intake in the gut and their connection with systemic indicators of malnourishment such as low blood glucose and body weight, demonstrating how the gut can be regulated by external and systemic stimuli. The crosstalk between gut and hepatic function was evident with several correlations between liver metabolites and intestinal gene expression data further supporting many of the hypotheses described in this paper.

Overall, this work helps to decipher key transcriptomic changes that result from a calorie and protein-restricted diet and how they are related to the observed phenotypes. This information and model will be useful moving forward to study this condition and dietary interventions.

## Data availability statement

The data presented in the study are deposited in the Sequence Read Archive (SRA) repository, https://www.ncbi.nlm.nih.gov/sra/, BioProject accession: PRJNA823693.

## Ethics statement

The animal study was reviewed and approved by the UC Davis Institutional Animal Care and Use Committee (IACUC).

## Author contributions

RP prepared the libraries, analyzed and interpreted the data, and wrote the manuscript. LG conducted the experiment that provided tissue samples for this study. EM conceived the project, obtained funding, developed the experiment design with LG and RP, and edited the manuscript. BH and BW were responsible for RNAseq library construction and library quality control. They also edited the manuscript. All authors contributed to the article and approved the submitted version.

## Funding

The provision of tissue samples for this study was supported by the Bill & Melinda Gates Foundation through the Grand Challenges Explorations initiative (OPP1067869) and the transcriptomic work was supported by a UC Davis Signature Research in Genomics Award.

## Conflict of interest

The authors declare that the research was conducted in the absence of any commercial or financial relationships that could be construed as a potential conflict of interest.

## Publisher's note

All claims expressed in this article are solely those of the authors and do not necessarily represent those of their affiliated organizations, or those of the publisher, the editors and the reviewers. Any product that may be evaluated in this article, or claim that may be made by its manufacturer, is not guaranteed or endorsed by the publisher.
