# Supplementary material for: Malnourishment affects gene expression along the length of the small intestine
Source: Front Nutr. 2022 Sep 2;9:894640. doi: 10.3389/fnut.2022.894640 (PMC9478944; doi:10.3389/fnut.2022.894640)
Supplement: Supplementary file 1 [file Data_Sheet_1.docx]

Supplementary Data, Table 1:

| **Index** | **Sequence 5’ to 3’** |
| --- | --- |
| **Universal Adapter** | AATGATACGGCGACCACCGAGATCTACACTCTTTCCCTACACGACGCTCTTCCGATCT |
| **Index 1** | GATCGGAAGAGCACACGTCTGAACTCCAGTCACATCACGATCTCGTATGCCGTCTTCTGCTTG |
| **Index 2** | GATCGGAAGAGCACACGTCTGAACTCCAGTCACCGATGTATCTCGTATGCCGTCTTCTGCTTG |
| **Index 3** | GATCGGAAGAGCACACGTCTGAACTCCAGTCACTTAGGCATCTCGTATGCCGTCTTCTGCTTG |
| **Index 4** | GATCGGAAGAGCACACGTCTGAACTCCAGTCACTGACCAATCTCGTATGCCGTCTTCTGCTTG |
| **Index 5** | GATCGGAAGAGCACACGTCTGAACTCCAGTCACACAGTGATCTCGTATGCCGTCTTCTGCTTG |
| **Index 6** | GATCGGAAGAGCACACGTCTGAACTCCAGTCACGCCAATATCTCGTATGCCGTCTTCTGCTTG |
| **Index 7** | GATCGGAAGAGCACACGTCTGAACTCCAGTCACCAGATCATCTCGTATGCCGTCTTCTGCTTG |
| **Index 8** | GATCGGAAGAGCACACGTCTGAACTCCAGTCACACTTGAATCTCGTATGCCGTCTTCTGCTTG |

Adapters used for mRNA library construction for Illumina platform sequencing.

Supplementary Data, Table 2:

Primers used for qPCR validation of DE genes.

| Gene | Amplicon size in cDNA (bp) | Efficiency | Reference |
| --- | --- | --- | --- |
| TBP  DPEP1  ME1  CXCL2  PCK1  SCD | 153  118  223  125  95  200 | 91.15  85  89.3  94  97.7  91.6 | Garas et al., 2017  Cao et al., 2004  Palma-Granados et al., 2019  Li et al., 2014  Qu and Ajuwon, 2018  Yu et al., 2013 |

Cao, H., Robinson, J. A. B., Jiang, Z., Melville, J. S., Golovan, S. P., Verrinder Gibbins, A. M. (2004). A high-resolution radiation hybrid map of porcine chromosome 6. *Anim. Genet.* 35, 367–378. doi:10.1111/j.1365-2052.2004.01161.x.

Garas, Lydia C., Cooper, Caitlin A., Dawson, Matthew W., Wang, Jane-Ling, Murray, James D., and Maga, Elizabeth A. (2017). Young pigs consuming lysozyme transgenic goat milk are protected from clinical symptoms of enterotoxigenic Escherichia coli infection. *J. Nutr.* 147, 2050–2059. doi:10.3945/jn.117.251322.

Li, Bin, Du, Luping, Sun, Bing, Yu, Zhengyu, Liu, Maojun, … He, Kongwang (2014). Transcription analysis of the porcine alveolar macrophage response to Mycoplasma hyopneumoniae. *PLoS One* 9, e101968. doi:10.1371/journal.pone.0101968.

Palma-Granados, P., Seiquer, I., Benítez, R., Óvilo, C., and Nieto, R. (2019). Effects of lysine deficiency on carcass composition and activity and gene expression of lipogenic enzymes in muscles and backfat adipose tissue of fatty and lean piglets. *Animal* 13, 2406–2418. doi:10.1017/s1751731119000673.

Qu, Huan and Ajuwon, Kolapo M. (2018). Cytosolic phosphoenolpyruvate carboxykinase is a response gene involved in porcine adipocyte adaptation to heat stress. *J. Anim. Sci.* 96, 1724–1735. doi:10.1093/jas/sky126.

Yu, Kaifan, Shu, Gang, Yuan, Fangfang, Zhu, Xiaotong, Gao, Ping, … Jiang, Qingyan (2013). Fatty acid and transcriptome profiling of longissimus dorsi muscles between pig breeds differing in meat quality. *Int. J. Biol. Sci.* 9, 108–118. doi:10.7150/ijbs.5306.

Supplementary Data, Table 3:

Differentially expressed genes in the duodenum between Mal and FF groups.

[DEG_duod.xls](file:///D:\Raquel\Desktop\Papers\Papers\Malnourishment\DEG_duod.xlsx)

Table 3: DESeq2 results from Salmon transcript abundance of DE genes in the duodenum between Mal and FF groups ordered by decreasing Padj. Log2FC- log2 fold change in abundance of the Mal group compared to FF; Padj - false discovery rate adjusted P-values.

Supplementary Data, Table 4:

Differentially expressed genes in the jejunum between the FF and Mal groups.

[DEG_Jej.xlsx](file:///D:\Raquel\Desktop\Papers\Papers\Malnourishment\DEG_Jej.xlsx)

Table 4: DESeq2 results from Salmon transcript abundance of DE genes in the jejunum between Mal and FF groups ordered by decreasing Padj. Log2FC- log2 fold change in abundance of the Mal group compared to FF; Padj - false discovery rate adjusted P-values.

Supplementary Data, Table 5:

Differentially expressed genes in the ileum between the FF and Mal groups.

[DEG_Ileum.xlsx](file:///D:\Raquel\Desktop\Papers\Papers\Malnourishment\DEG_Ileum.xlsx)

Table 5: DESeq2 results from Salmon transcript abundance of DEG in the ileum between Mal and FF groups ordered by decreasing Padj. Log2FC- log2 fold change in abundance of the Mal group compared to FF; Padj - false discovery rate adjusted P-values.

Supplementary Data, Figure 1:

Reactome pathway analysis in the ileum of full-fed and malnourished pigs. A) Reactome pathway analysis using the downregulated DE entrez gene ID in humans. Edges connect associated pathways. Size and color of nodes represent the number of genes up or downregulated and padj values, respectively. B) Reactome analysis of the upregulated genes in the ileum of Mal compared to FF piglets. Bar length represents the counts of genes DE in each pathway, colors represent the padj value.


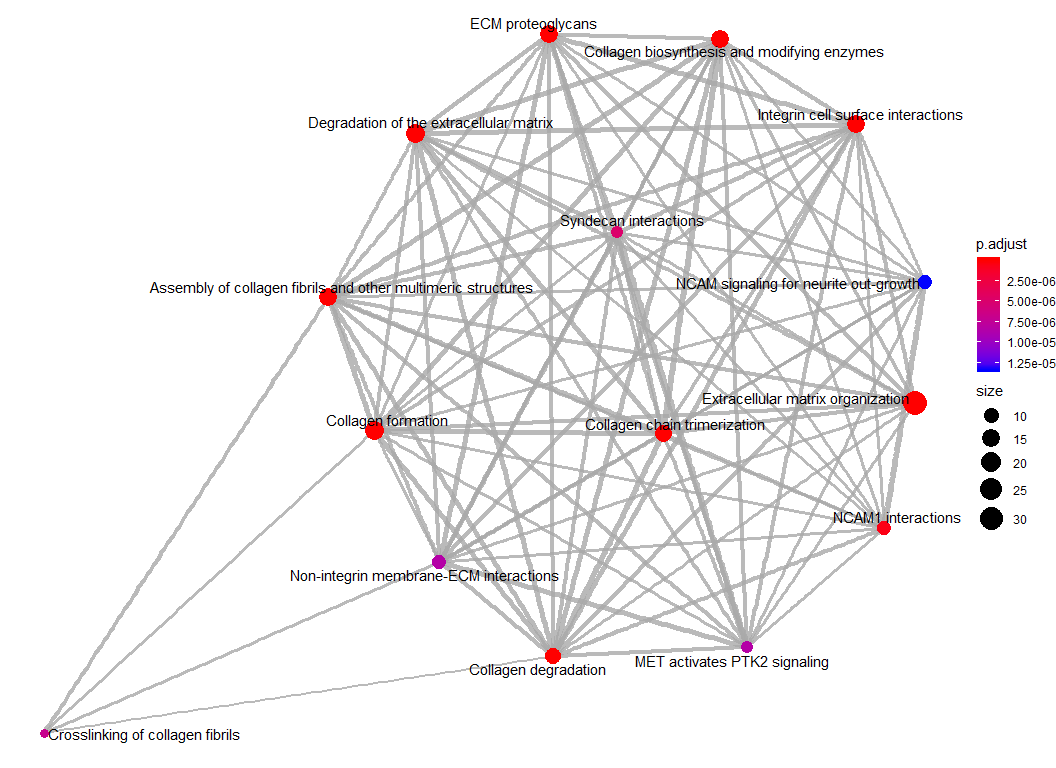


A


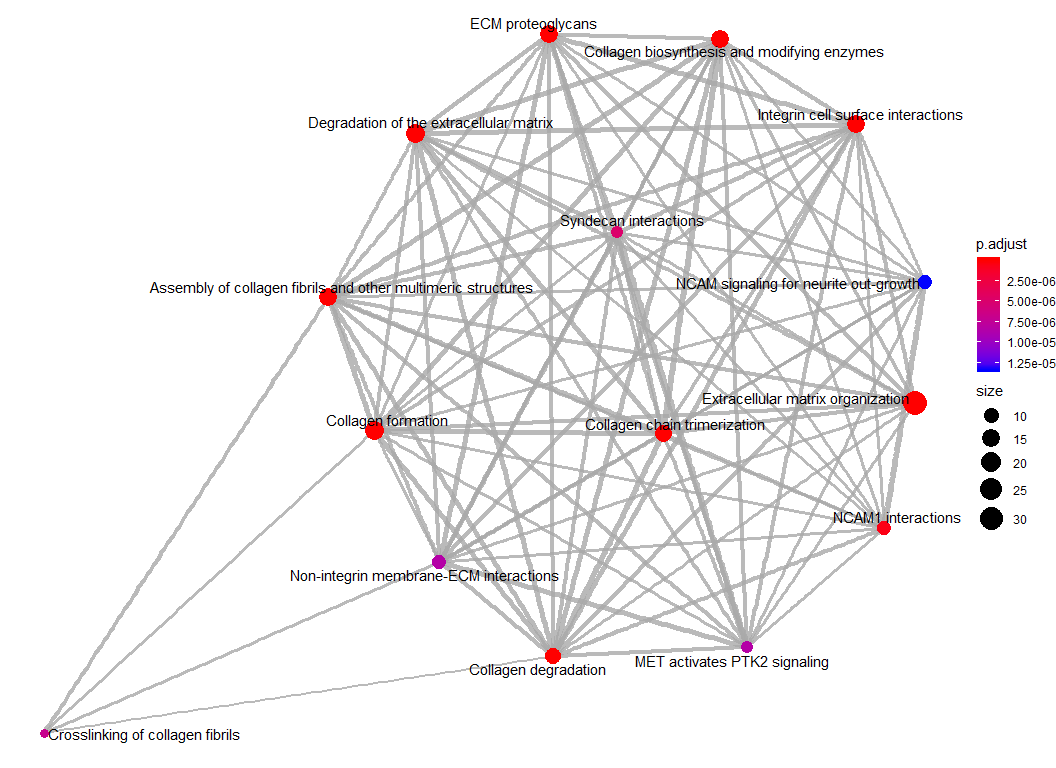


B


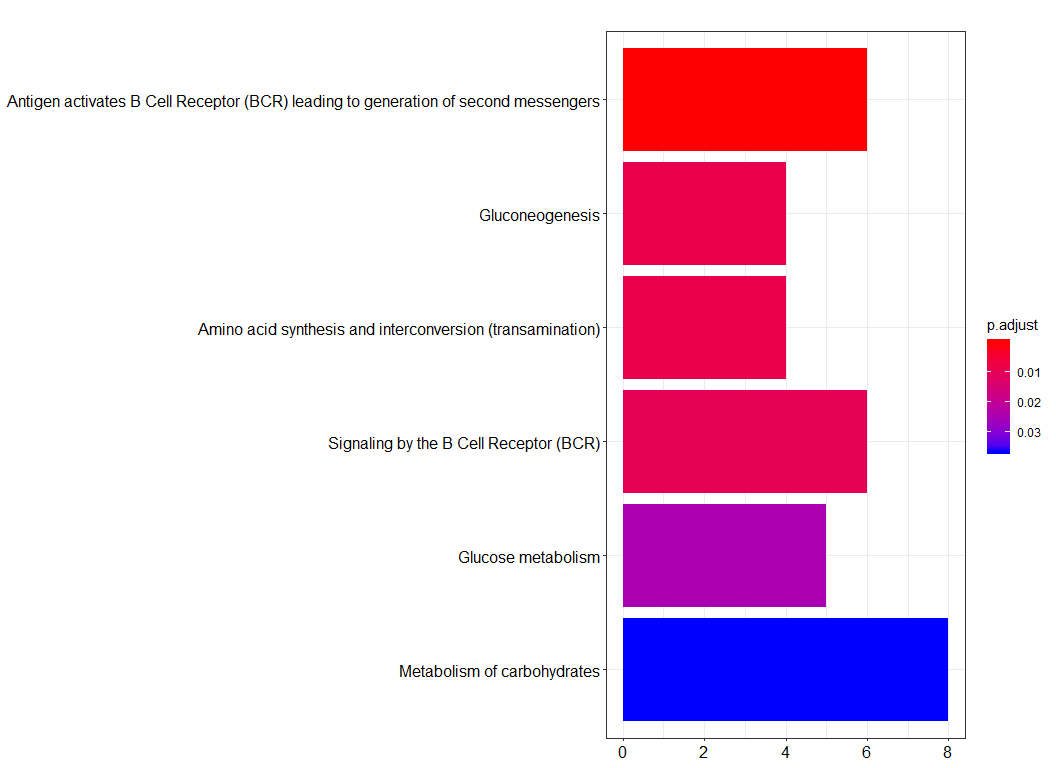

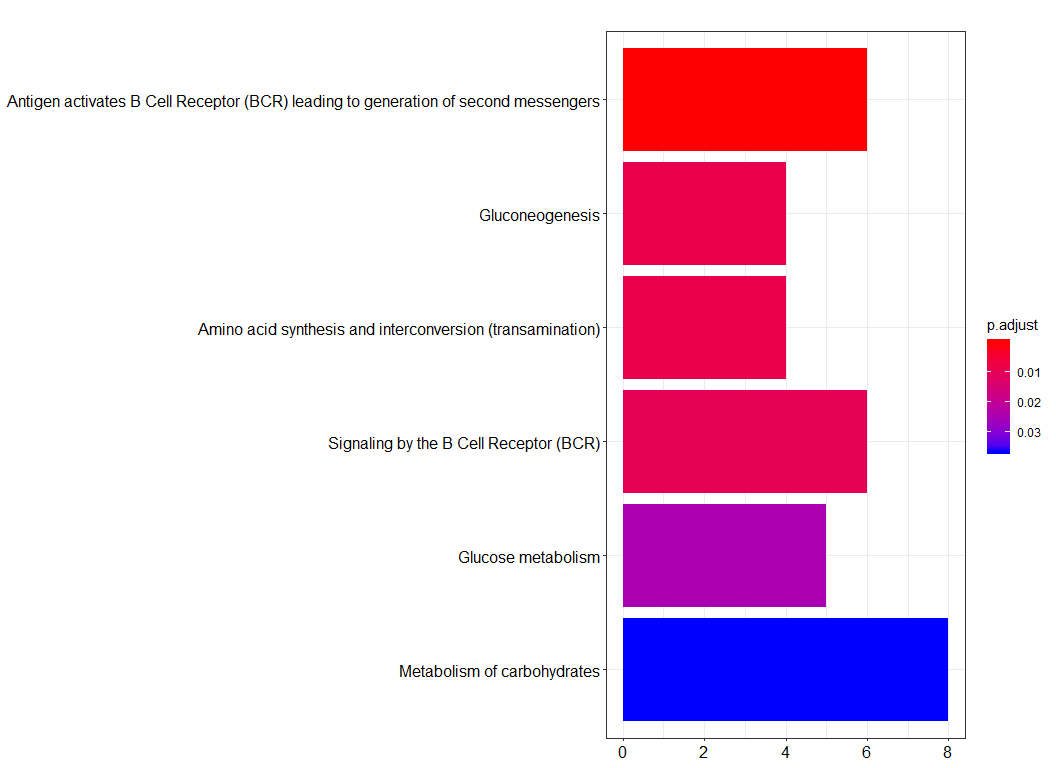

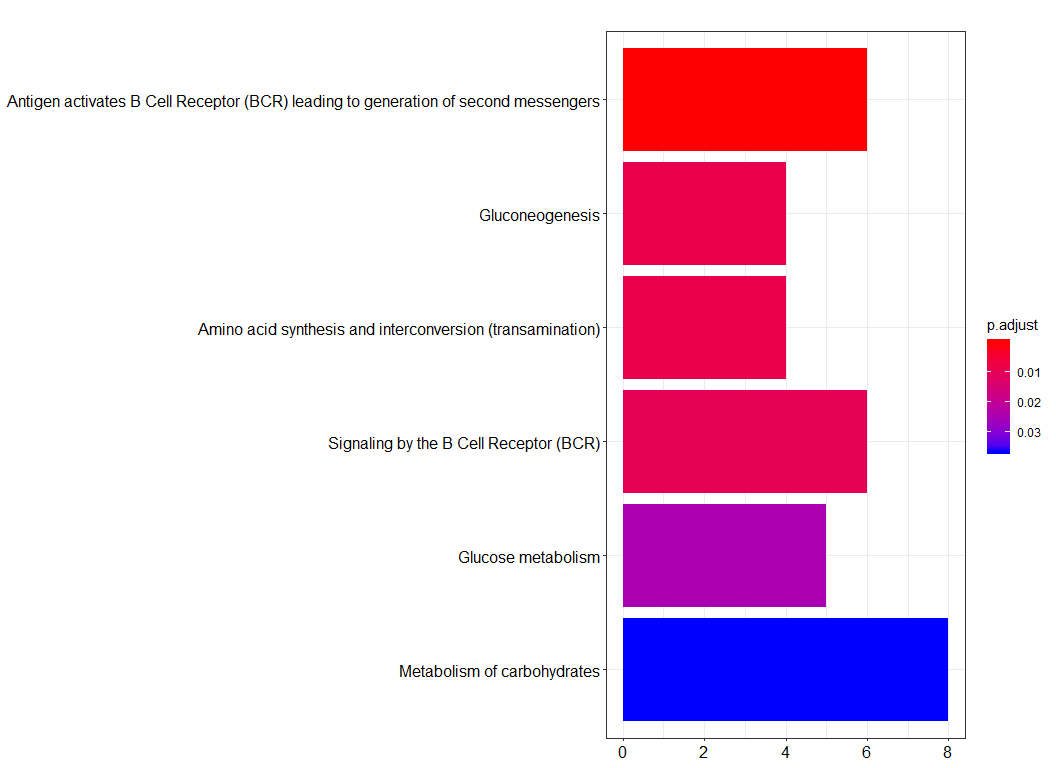

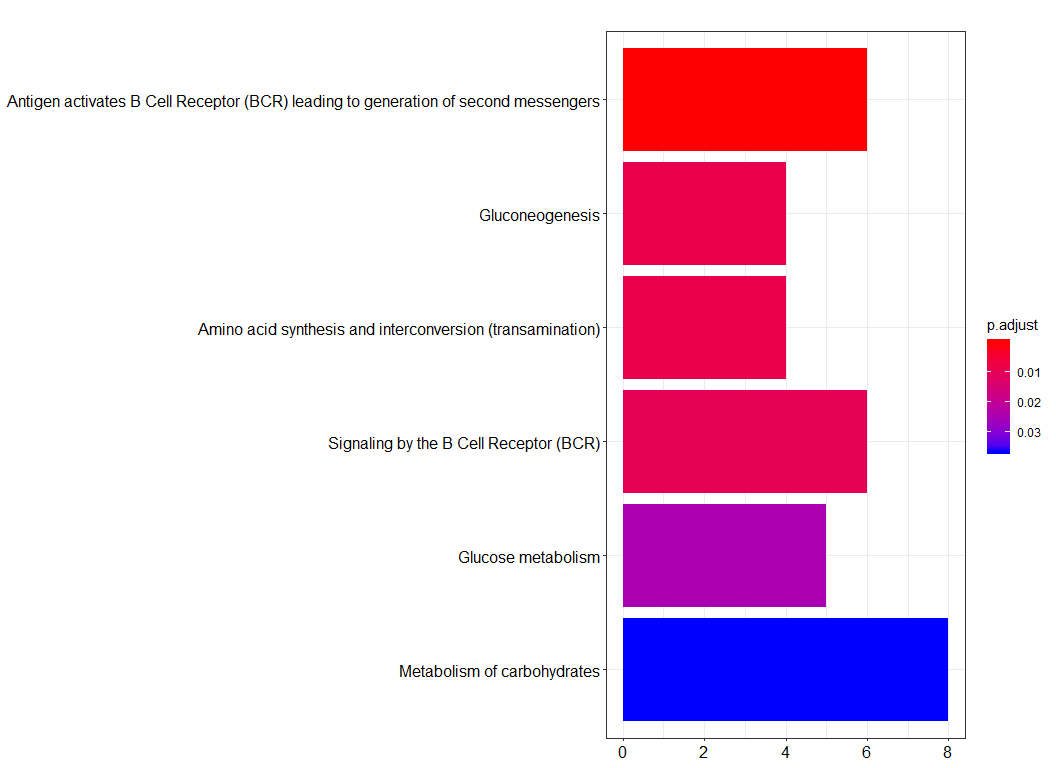

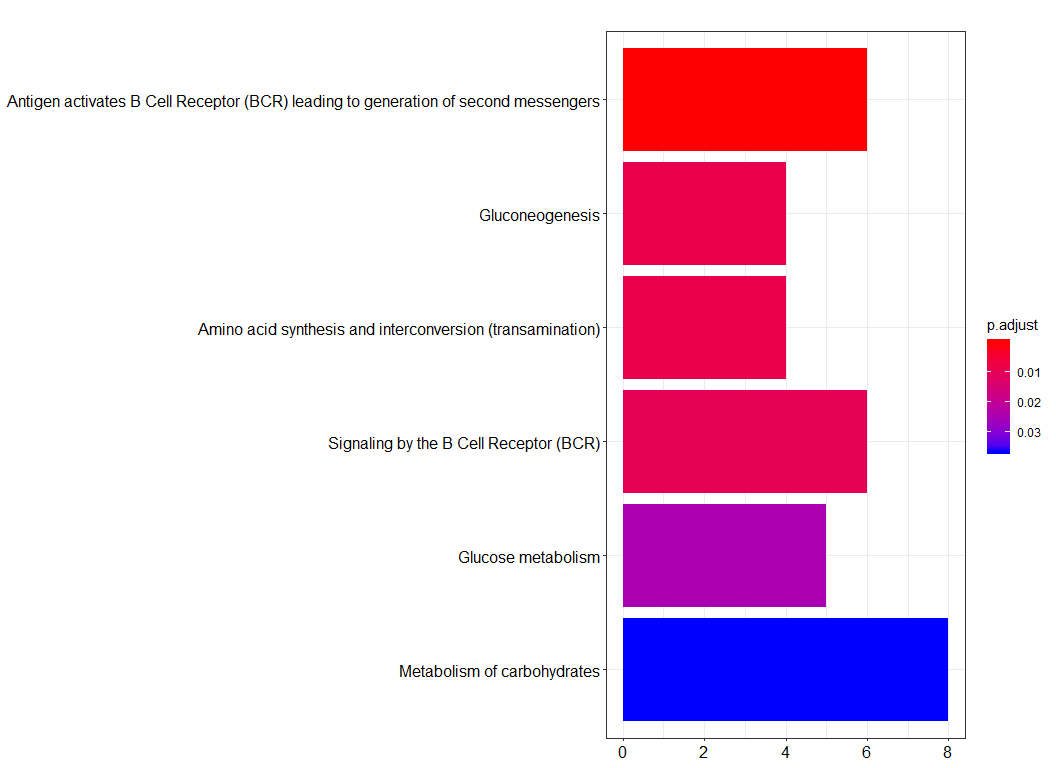

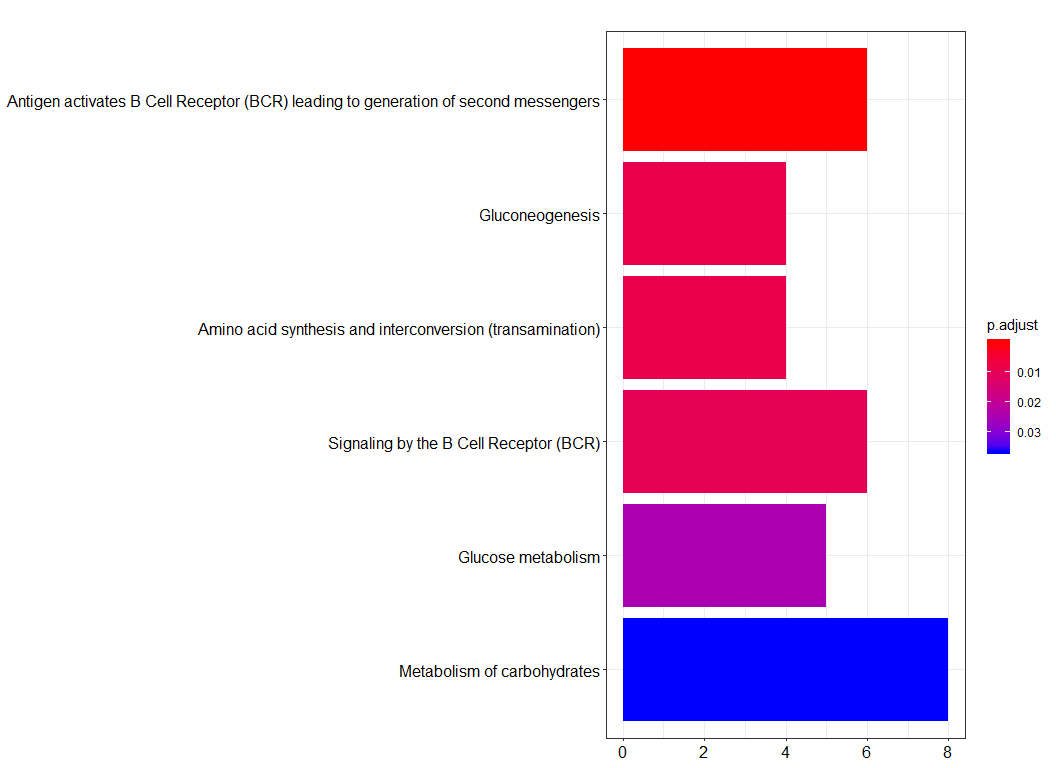

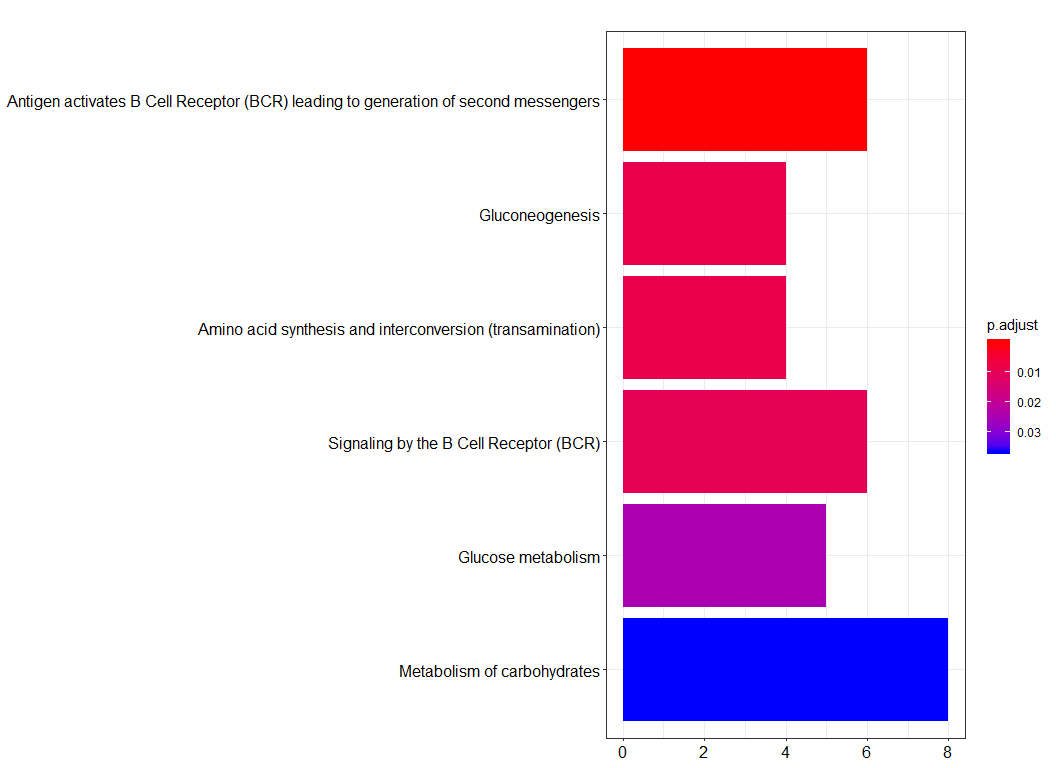

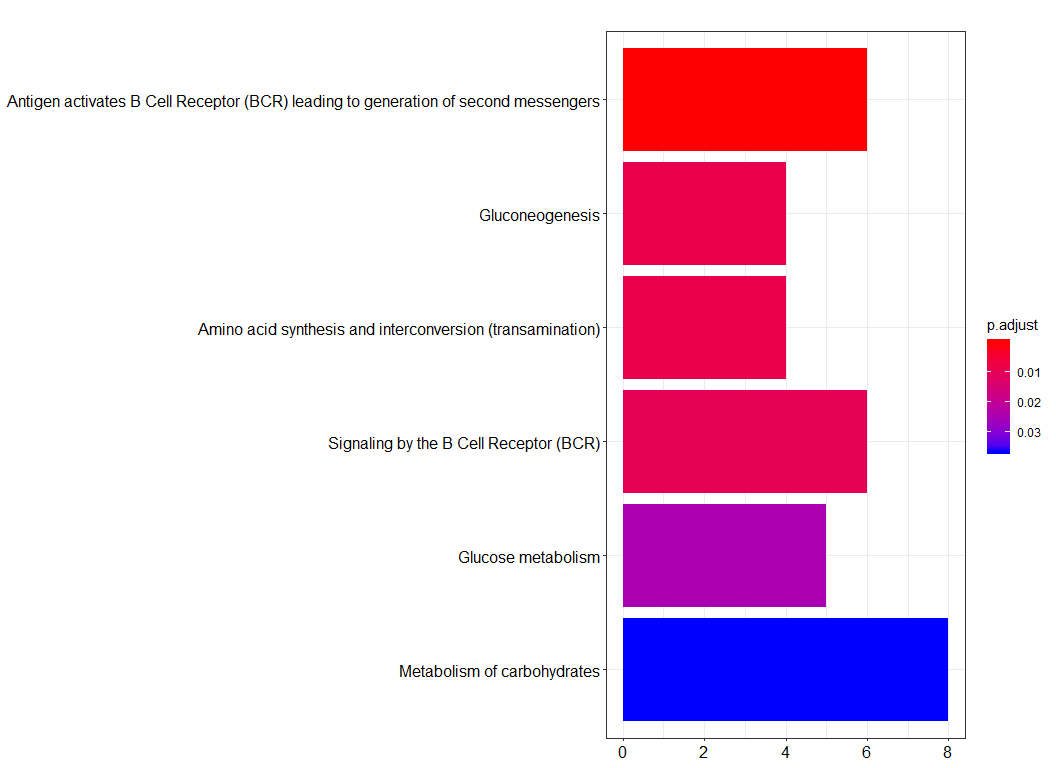

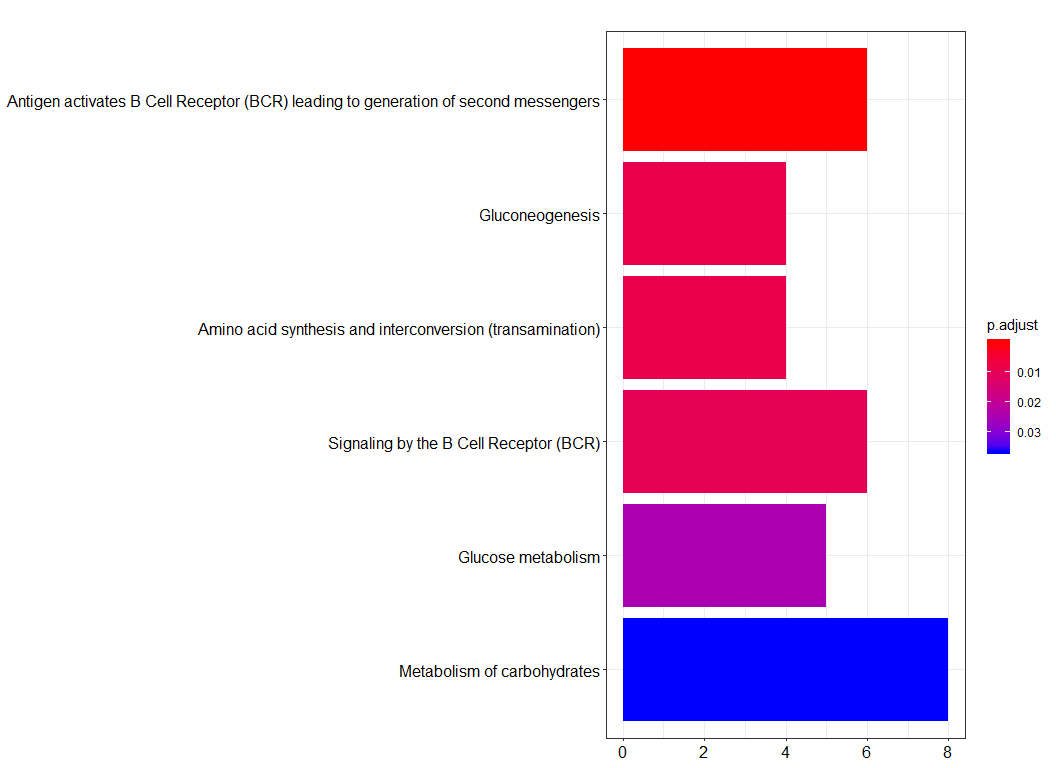

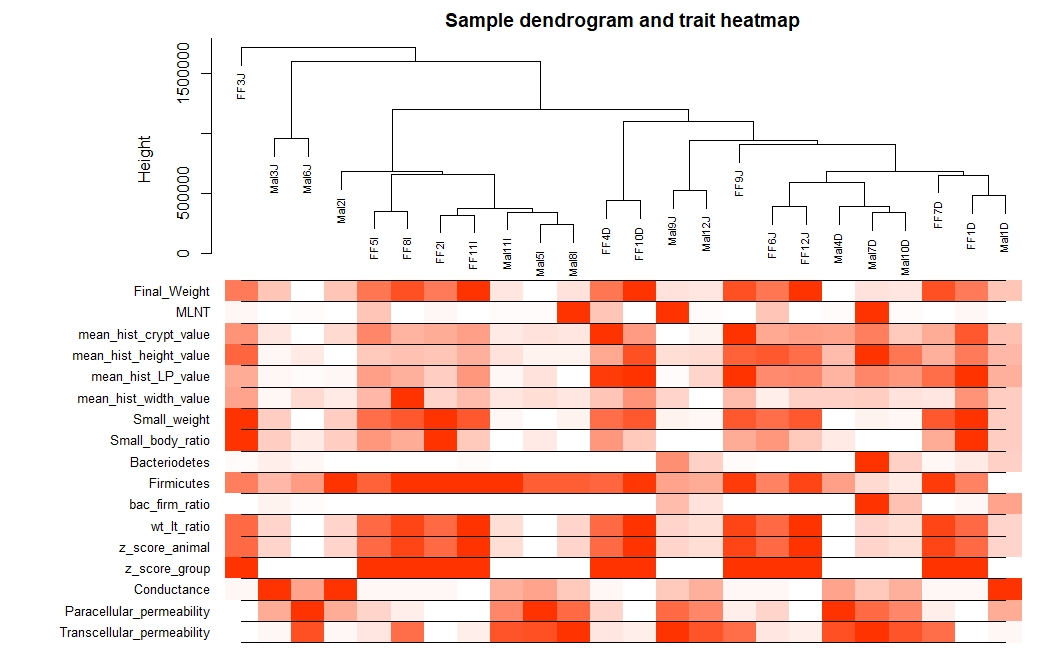


Supplementary Data, Figure 2:

Sample dendrogram and trait heatmap for the full-fed and malnourished groups. Dendrogram of intestinal samples from full-fed (FF) and malnourished animals (Mal) and the heatmap of the phenotype data of the animals. The phenotypic data from FF animals is highlighted with back boxes. Values of the trait indicated by the row is displayed in a gradient from low (white) to high (red).

Supplementary Data, Figure 3:

Consensus modules-trait relationship across groups. Consensus modules built with samples from the FF and Mal groups are identified as colors on the y-axis and traits of both FF and malnourished animals are on the x-axis. Rounded p-values < 0.05 are displayed in the heatmap, color indicative of positive (red) or negative (green) correlation between the gene module and phenotype. The block of modules associated with the histological data is highlighted with a black box.


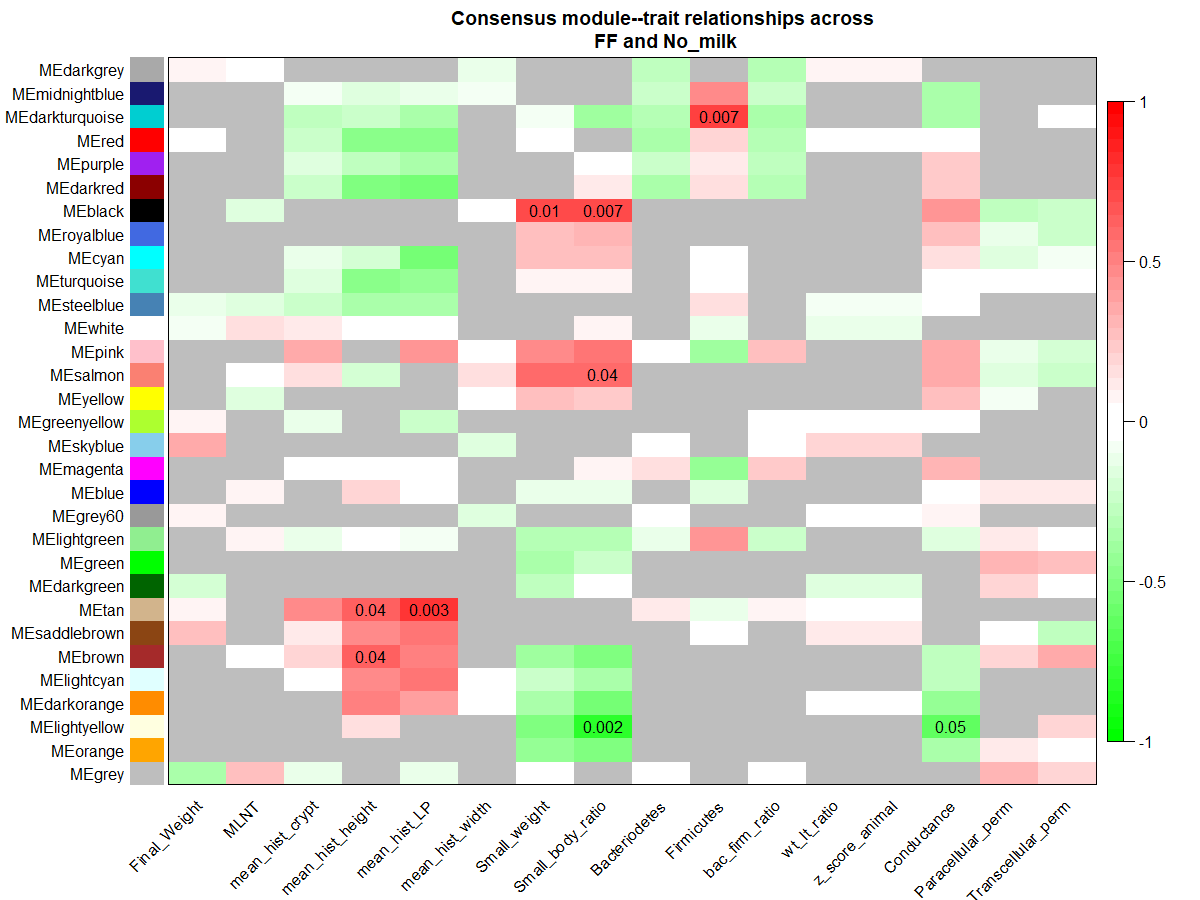


Supplementary Data, Table 6:

| **Consensus modules** | **Trait** | **P-value** | **N. genes** |
| --- | --- | --- | --- |
| **Lightyellow** | Small intestine/body weight | 0.0016 | 225 |
| **Tan** | Lamina Propria | 0.0029 | 368 |
| **Black** | Small intestine/body weight | 0.0068 | 565 |
| **Darkturquoise** | Firmicutes | 0.0073 | 139 |
| **Black** | Small intestine weight | 0.01 | 565 |
| **Tan** | Villi height | 0.036 | 368 |
| **Salmon** | Small intestine/body weight | 0.036 | 359 |
| **Brown** | Villi height | 0.044 | 951 |
| **Lightyellow** | Conductance | 0.045 | 225 |

Significant consensus module - trait relationships across groups.

Table 6: Most significant relationships between consensus modules and traits across all animals. N. genes is the number of genes in the module.

Supplementary Data, Table 7:

| **Consensus modules** | **Trait** | **P-value** | **N. genes** |
| --- | --- | --- | --- |
| **Lightcyan** | Lamina propria | 2.21e-06 | 285 |
| **Red** | Firmicutes | 1.24e-05 | 621 |
| **Tan** | Lamina Propria | 9.74e-05 | 368 |
| **Tan** | Firmicutes | 0.00011 | 368 |
| **Lightcyan** | Villi height | 0.00013 | 285 |
| **Grey** | Small intestine/body weight | 0.00014 | 1349 |
| **Cyan** | Firmicutes | 0.00026 | 302 |
| **Lightyellow** | Small intestine/body weight | 0.00035 | 225 |
| **Grey** | Conductance | 0.00062 | 1349 |

Most significant consensus module-malnourished traits relationships.

Table 7: Most significant relationships between consensus modules and traits from malnourished animals. N. genes is the number of genes in the module.

Supplementary Data, Table 8:

Reactome analysis of DE genes in the light-cyan consensus module.

| **Term** | **Description** | **Counts** | **P-value** | **Genes** |
| --- | --- | --- | --- | --- |
| **R-HSA-425381** | Bicarbonate transporters | 2 | 0.011 | AHCYL2/SLC4A4 |
| **R-HSA-375276** | Peptide ligand-binding receptors | 4 | 0.014 | CXCL2/PENK/  CCR10/SST |
| **R-HSA-196741** | Cobalamin (Cbl,  vitamin B12) transport  and metabolism | 2 | 0.015 | TCN1/CBLIF |
| **R-HSA-418594** | G alpha (i) signalling  events | 5 | 0.015 | CXCL2/RBP4/  PENK/CCR10/SST |
| **R-HSA-373076** | Class A/1 (Rhodopsin-like receptors) | 4 | 0.043 | CXCL2/PENK/  CCR10/SST |
| **R-HSA-380108** | Chemokine receptors  bind chemokines | 2 | 0.046 | CXCL2/CCR10 |

Supplementary Data, Figure 4:

Plot and correlation matrix of samples from A) duodenum, B) jejunum and C) ileum in the spatial representation of individual datasets. Correlation matrix between DE genes in the transcriptome (mRNA_DE), microbiota relative abundance (data_16S) and liver metabolites (Metabolites) between full-fed (FF, blue) and malnourished animals (Mal, orange)


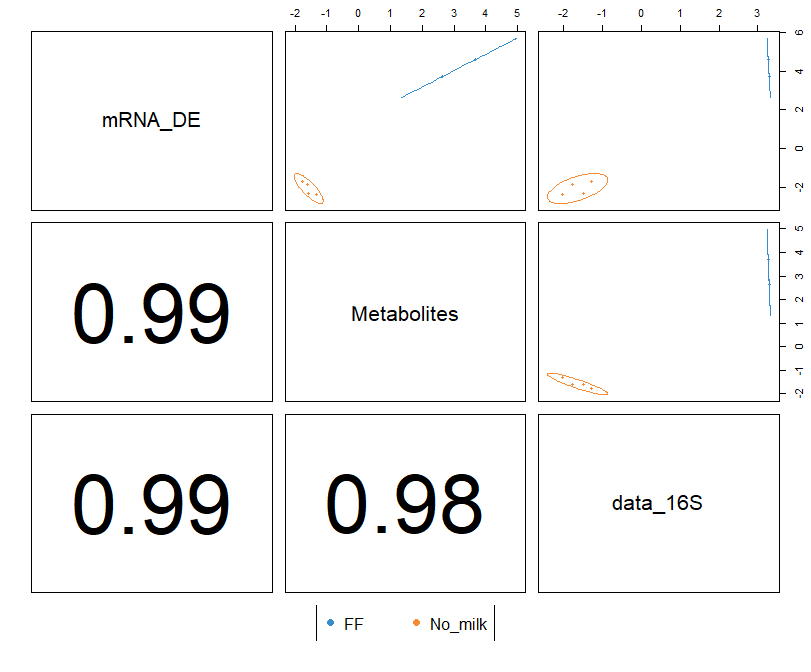


B

B


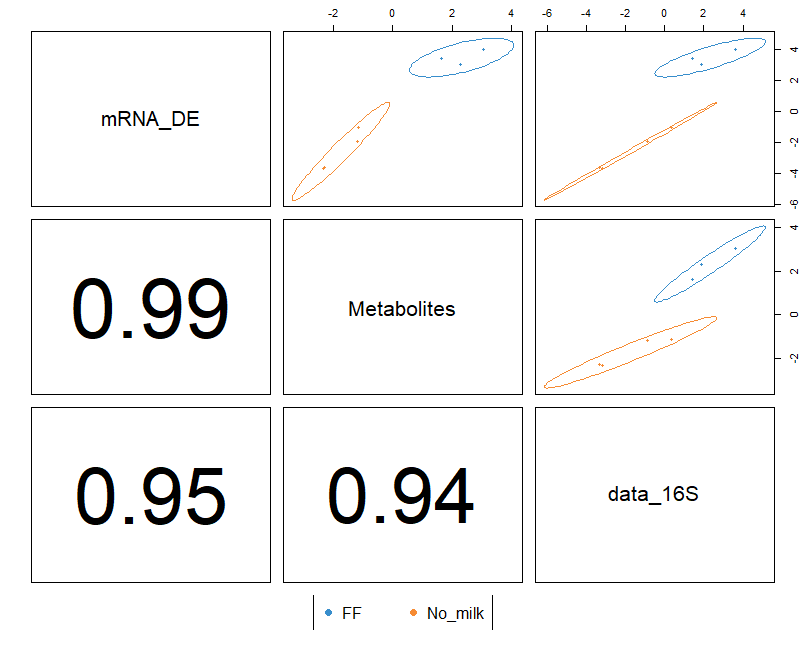


A

A


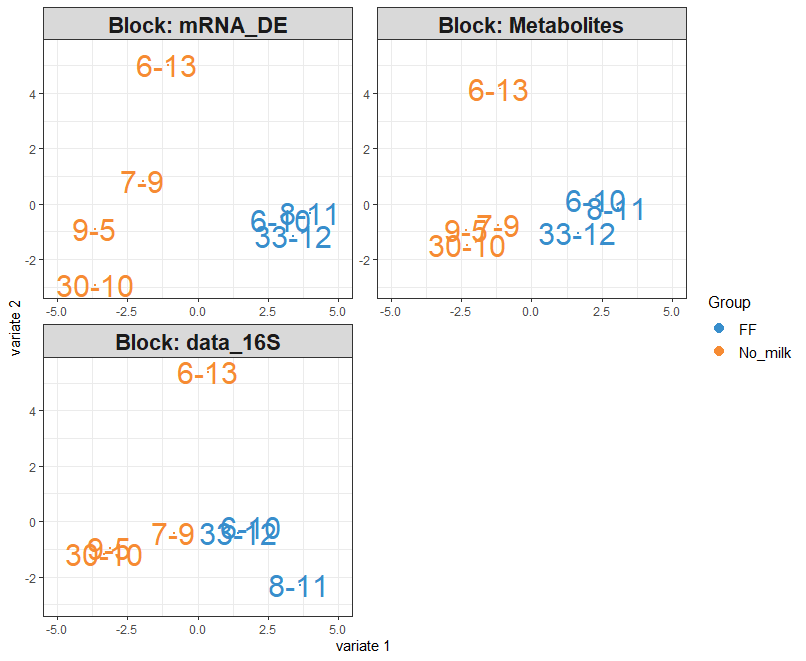


FF

Mal


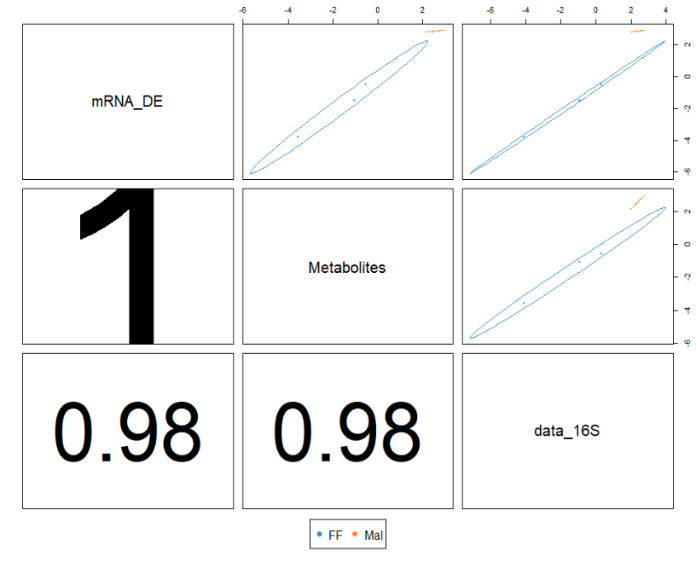


C

A

Supplementary Data, Figure 5:

Circle plot of transcriptome, intestinal content microbiota and liver metabolome data in jejunum. Circle plot of genes (mRNA_DE), intestinal content microbiota (data16S) and metabolites positioned according to the latent components 1 and 2 output of DIABLO analysis for jejunum.


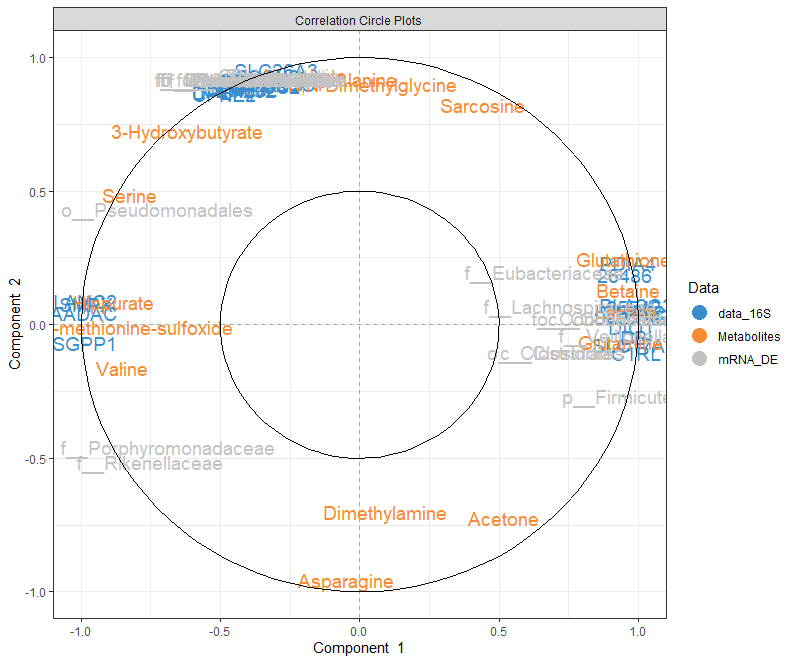

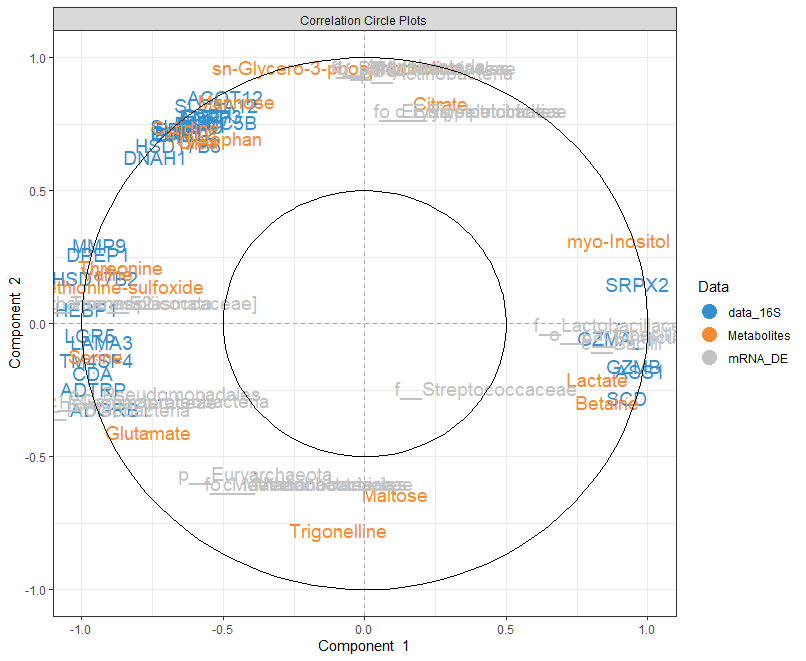

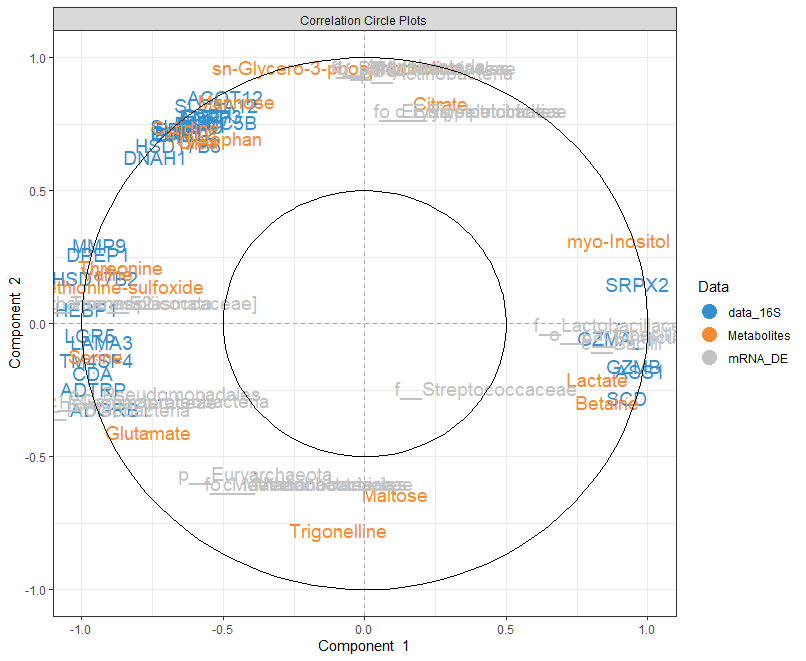


Supplementary Data, Table 9:

Coordinates of the variables overlapping (correlated with) Betaine in the jejunum.

| **Name** | X | Y | Dataset |
| --- | --- | --- | --- |
| **Glutamine** | 0.938375823 | -0.063991284 | Metabolites |
| **c__Coriobacteriia** | 0.945872379 | 0.025190701 | data_16S |
| **o__Coriobacteriales** | 0.945872379 | 0.025190701 | data_16S |
| **f__Coriobacteriaceae** | 0.945872379 | 0.025190701 | data_16S |
| **p__Firmicutes** | 0.946030999 | -0.267099498 | data_16S |
| **Glutathione** | 0.950935756 | 0.247199321 | Metabolites |
| **26486** | 0.952523599 | 0.185790178 | mRNA_DE |
| **Lactate** | 0.957549513 | 0.052251316 | Metabolites |
| **PDIA4** | 0.966324302 | 0.229851810 | mRNA_DE |
| **Betaine** | 0.966450731 | 0.130750683 | Metabolites |
| **SLC7A5** | 0.971644787 | 0.029762866 | mRNA_DE |
| **DIO1** | 0.974424864 | -0.006703771 | mRNA_DE |
| **35542** | 0.984863630 | 0.065028978 | mRNA_DE |
| **SLC17A9** | 0.987501469 | -0.077594828 | mRNA_DE |
| **SHAS2** | 0.987988187 | 0.046761678 | mRNA_DE |
| **C1RL** | 0.994019764 | -0.106984297 | mRNA_DE |
| **PLA2G3** | 0.994396311 | 0.073844665 | mRNA_DE |
| **f__Veillonellaceae** | 0.994894917 | -0.038283722 | data_16S |
| **SCD** | 0.996125760 | 0.052830589 | mRNA_DE |
| **DBI** | 0.998477848 | -0.045273912 | mRNA_DE |
